# Towards a category theory approach to analogy: Analyzing re-representation and acquisition of numerical knowledge

**DOI:** 10.1371/journal.pcbi.1005683

**Published:** 2017-08-25

**Authors:** Jairo A. Navarrete, Pablo Dartnell

**Affiliations:** 1 Departamento de Ciencias Básicas, Universidad del Bío-Bío, Av. Andrés Bello 702, Chillán, Chile; 2 Centro de Modelamiento Matemático (CMM), Centro de Investigación Avanzada en Educación (CIAE) de la Universidad de Chile, Periodista José Carrasco Tapia Nº 75, Santiago, Chile; University of Minnesota, UNITED STATES

## Abstract

Category Theory, a branch of mathematics, has shown promise as a modeling framework for higher-level cognition. We introduce an algebraic model for *analogy* that uses the language of category theory to explore analogy-related cognitive phenomena. To illustrate the potential of this approach, we use this model to explore three objects of study in cognitive literature. First, (a) we use *commutative diagrams* to analyze an effect of playing particular educational board games on the learning of numbers. Second, (b) we employ a notion called *coequalizer* as a formal model of re-representation that explains a property of computational models of analogy called “flexibility” whereby non-similar representational elements are considered matches and placed in structural correspondence. Finally, (c) we build a formal learning model which shows that re-representation, language processing and analogy making can explain the acquisition of knowledge of rational numbers. These objects of study provide a picture of acquisition of numerical knowledge that is compatible with empirical evidence and offers insights on possible connections between notions such as relational knowledge, analogy, learning, conceptual knowledge, re-representation and procedural knowledge. This suggests that the approach presented here facilitates mathematical modeling of cognition and provides novel ways to think about analogy-related cognitive phenomena.

## Author summary

Analogy is claimed to be the core of human cognition due to its pervasive involvement in phenomena such as language, reasoning and learning. However, this phenomenon has mainly been studied in isolation through computational methods, which has made it difficult to appreciate its different roles in diverse cognitive phenomena. Recent studies have signaled that abstract concepts from category theory are able to describe constructions carried out by the human mind, thus presenting this formal theory as a general framework to study cognition. Our contribution here is to provide a model of analogy that bridges a gap between the formal notions of category theory and the psychological notions of cognition. We illustrate the usefulness of this approach by using our model to represent and analyze three different cognitive phenomena. Besides showing that some abstract mathematical concepts can describe concrete cognitive notions such as re-representation, learning, conceptual knowledge, and procedural knowledge, the process of mathematical modeling is presented here as an alternative for outlining relations between cognitive notions and for developing novel conceptualizations of the involved cognitive phenomena.

## Introduction

More than five decades ago, a formal notion of “isomorphism” was used to define “representations” that sustained measurement theory and models of cognitive systems [1, 2]. Afterwards, it was proposed to describe how certain processes in a cognitive symbol system are able to reflect corresponding processes in an environment [3]. More recently, this formal notion was proposed to describe associations between “mental representations” and “environments” as a general framework to approach cognition [4, 5]. In regard to analogy making, this notion satisfied the *structural consistency principle* proposed by Dedre Gentner at the core of analogy [6, 7], but it was pointed out that the constraints imposed by this formalism would be too strong to be useful since isomorphisms identify entities that share the exact same internal structure. This notion was then weakened as a framework for modeling analogies because “the kinds of analogies of psychological interest, virtually never have the structure of a valid isomorphism” [5, p. 300].

But the proposal of similar but more flexible notions allowed novel conceptualizations of analogies and other cognitive phenomena. For example, the notion of structure preserving mappings between two domains—called *morphisms*—motivated proposals such as the *quasi-homomorphism*, namely a morphism endowed with “exceptions” where the map would not preserve the structure [4]. Additionally, the notion of a *local homomorphism* is a partial mapping that preserves the structure only where the map is defined i.e. a map that does not preserve the structure of the entire object [8]. These ideas have proven valuable as cognitive modeling tools, but they still have not taken full advantage of the richness provided by the notion of morphisms.

There are claims pointing to the advantages of studying cognitive phenomena through a branch of mathematics called Category Theory which is a theory of structure based on formal notions such as *morphisms, limits, colimits, products, adjuntions* and other concepts developed out of a need to formalize commonalities between various mathematical structures [9–11]. It has been pointed out that the human mind has the ability to carry out a large number of constructions that seem so universal that they must be somehow severely constrained. And that these constraints might well be adequately formalized by the notions proposed by category theory which describe the natural constraints of mathematical constructions [12]. Furthermore, category theory has been proposed as the foundation for a theory of cognitive developmental stages [3, 13]

Along this path, it is emphasized that the use of category theory could lead to developing new and fruitful analyses of classical cognitive notions [13]. For example, *systematicity* is the feature of human cognition whereby cognitive capacity comes in groups of related behaviours. The properties of this phenomenon cannot be explained neither by classical nor connectionist theories because these theories must make ad-hoc assumptions on their respective representations of knowledge i.e. grammars and neural networks [14]. However, when it is examined under a categorical approach, by using notions such as products and adjunctions, the systematicity properties turn out to be *uniquely* determined. This approach thus explains systematicity without making ad-hoc assumptions [15]. Further support for the potential of category theory as an analytic tool is provided by similar studies of cognitive phenomena [16–19].

On another aspect, category theory seems to be also useful as a language for formulating cognitive processes [20]. Formal notions such as limits and colimits have been used in models of reasoning about space and time [21, p. 25], in semantic models for neural networks [22], and in theories about brain’s spatial representation [23, 24]. Similarly, the notion of morphism has been used to describe structure preserving paths between artificial perceptions [25, 26], and to formalize the functional relation between man-machine interfaces and their machine-functionality (in approaches to user interface development) [27, 28].

There are various applications of category theory to research cognitive psychology [12, 15–18] but applications of category theory to research analogy have been almost non-existent (see however [19, 26]). This is odd because category theory has been used extensively in computer science for the analysis of computation [29–31], and computationalism has been the main tool to research analogy. Our goal here is to give a first step into developing a category theory-based approach for analyzing and modeling cognitive phenomena directly related to analogy.

In the present study, we introduce a simple mathematical model of analogy (MMA) and illustrate how its application allows using notions of category theory into studying cognition: (1) We use the model as a device to elaborate a theory able to describe the effects of playing board games on children’s learning of mathematics [32, 33]. (2) We use the formal notion of a *coequalizer* to explain a property of symbolic models of analogy called *flexibility* whereby non-similar representational elements are considered matches and placed in structural correspondence during the analogical comparison [34]. (3) We formalize a frequently used method for teaching fractions to children thus obtaining a formal learning model that suggests that the abilities of re-representation, language processing and analogy making can explain the acquisition of knowledge of rational numbers.

The analyses performed on the aforementioned three objects of study offer insight on the role of analogy in these cognitive phenomena while illustrating the potentialities of applying category theory into the study of cognition. In these analyses, we show how some abstract structures provided by category theory can help us to arrange cognitive notions into formal theories capable of explaining, analyzing and organizing cognitive material. Above all, these three objects of study illustrate how this approach can help in developing novel ways to think about cognitive phenomena. In this way, we argue here that the MMA (see Definition 0.3 below) provides a convenient bridge between the formal notions provided by category theory and the psychological notions necessary to develop theories of cognition.

### Analogy

Analogy enables humans to gain understanding of unknown structures (*target domains*) by using knowledge of previously known structures (*source domains*). Evidence supporting this point of view, together with the observation that analogy is pervasive in language and thought, suggests a key role for analogical processes at the core of human cognition [35–40]. This suggests that analogy plays a key role in diverse fields such as linguistics, psychology, cognitive science, education and artificial intelligence [5, 6, 41–45] among others.

The study of analogy has been mainly done through computational models and simulations of the phenomenon. Some of these models are called *symbolic* because they describe domains of knowledge as sets of formulas of symbolic languages [7, 46]. Other models are called *connectionist* because they represent semantic knowledge through neural networks and distributed representations [43, 47, 48]. If these two features are present in a computational model, it is called *hybrid* because it represents knowledge through the integration of syntax and semantics [42, 49]. A review of these families of models has been presented in [34] and hence we present here only a short review of analogy models that can be regarded as the predecessors of the formal model presented in the next section.

**Fig 1:**
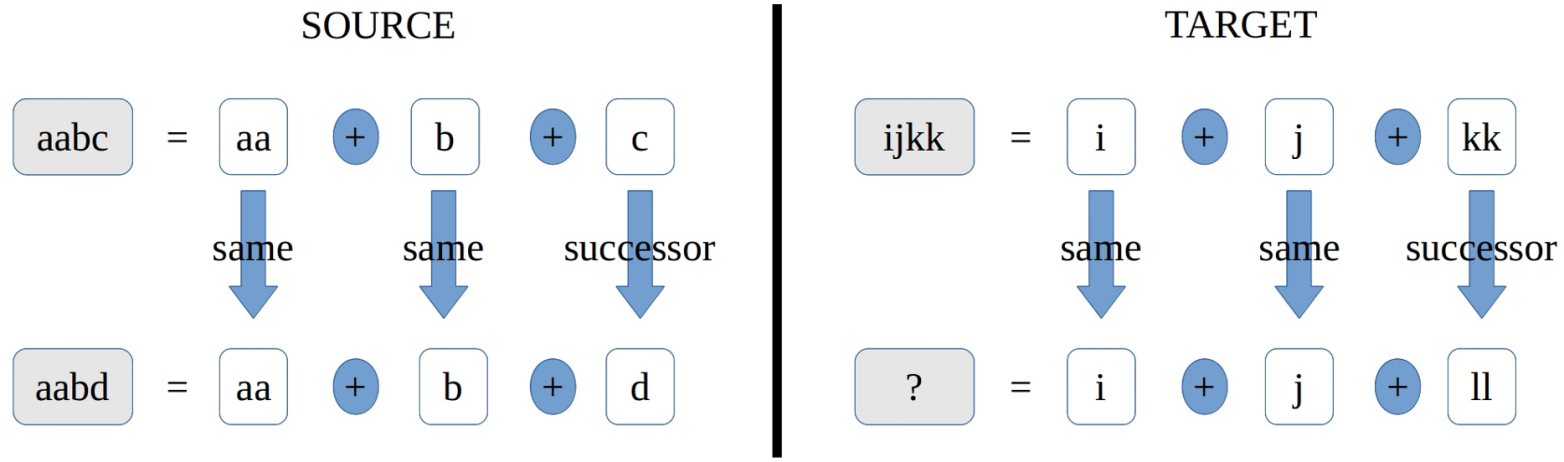
The unknown *ijll* is obtained by constructing representations of the source and target having the exact same structure. In the picture, such structure is represented by the operators given by the blue arrows and the blue circles.

COPYCAT solves proportional analogies in a domain of strings of characters: Its authors would ask “suppose the letter-string *aabc* were changed to *aabd*; how would you change the letter-string *ijkk* in ‘the same way’? [42]. To analyze this kind of problems, copycat has perceptual agents named *codelets* which, by using a stochastic method called simulated annealing, combine the primitives stored in its *slipnet* (the letters *a*, *b*, *c*, *d*, …, *z*) using operators such as “succesor”, “predecessor”, “same” and others that permit it to construct an internal representation of the problem (see also TABLETOP in [49]). In our example, the *codelets* choose the answer *ijll* by generating representations such as the one described by Fig 1. Since copycat uses a non-deterministic algorithm to solve the combinatorial problem, other answers such as *ijkl* or *hjkk* are also given.

Around the same time, a formal treatise of analogy was proposed as the basis of a theory of cognition inspired in algebraic concepts [40]. To represent knowledge, the notion of *conceptual network* is defined as a *finitely generated algebra* i.e. a set of objects along with some operations defined on that set. An analogy from conceptual network ***A*** to conceptual network ***B*** is called a *cognitive relation* which is defined as a subset of the product ***A*** × ***B***.

**Fig 2:**
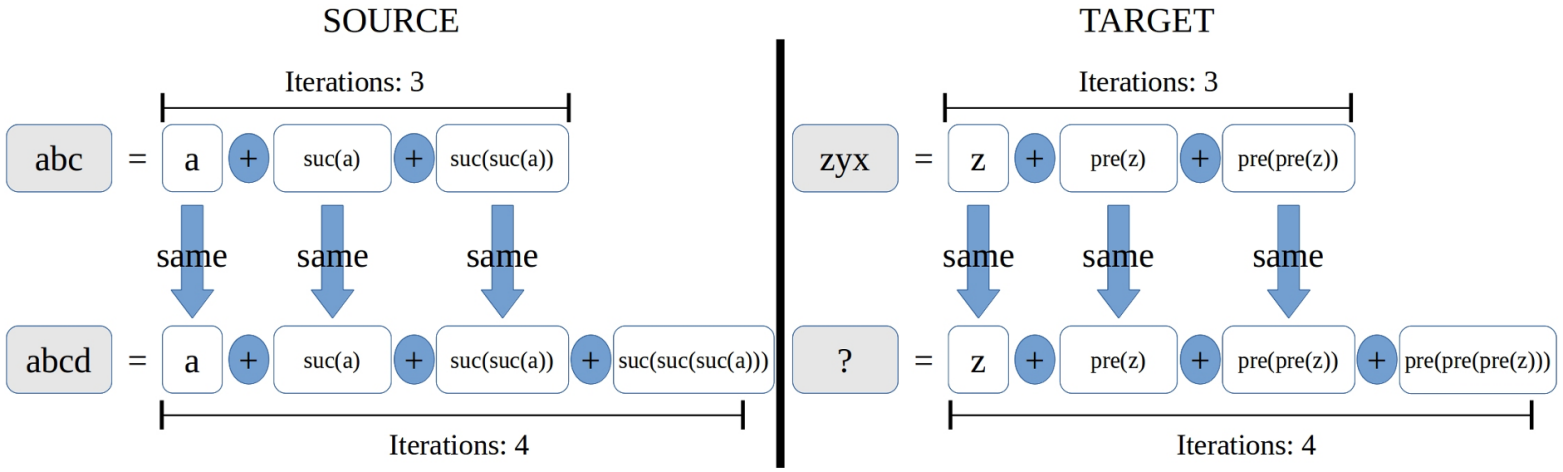
The unknown *zxyw* is obtained by constructing representations of the source and target that have identical structure. In the picture, such structure is represented by the operators given by the blue arrows, the blue circles and the times of iteration. Notice that it is necessary to establish an associaton between source*’*s *suc* (standing for *succesor*) and target’s *pre* (standing for *predecessor*).

These ideas were then used to solve proportional analogies in string domains: Dastani and Indurkhya [8] developed a computational model using the algebra determined by the characters *a*, *b*, *c*, *d*, …, *z* and some operators (or *gestalts*) such as “iteration”, “succesor”, “symmetry” and “alternation”. Problems such as *abc*: *abcd*::*zyx*:? were solved by computing *local homomorphisms* i.e. partial maps preserving the algebraic structure only in the subset where the partial map is defined. For example, to solve the mentioned problem, the core algorithm of this model capitalizes on Indurkhya’s ideas by: (1) generating subalgebras *A*_1_ and *A*_2_ such that *abc*, *abcd* belong to *A*_1_ and *zyx* belongs to *A*_2_, (2) generating identical representations for *abc* and *zyx* (see Fig 2 to see what “identical” means) and (3) generating a *local homomorphism* *h*: *A*_1_ → *A*_2_ that satisfies *h*(*abc*) = *zyx*. This last step entails the solution *h*(*abcd*) = *zyxw*.

The reviewed models blend together representational processes and analogy-making processes by using an algebraic technique: fixed primitives are combined to generate representations of the relevant domains. Their algorithms are designed to build representations of source and target that share the same structure. This way, the analogy is constructed by forcible interaction of processes of analogy-making and representation. This short review sets an appropriate context to introduce our formal model of analogy because the model proposed in the next section can be thought as an abstraction of these computational models. The mathematical definitions introduced below will be exemplified through computing solutions for analogy problems similar to the ones depicted in the figures above.

## Model

### An algebraic model for analogy

Let us first introduce some mathematical tools to formalize concepts such as the source, the target and the analogical map. These key components of the model will be illustrated through formalizing the two following proportional analogy problems borrowed from [8]:

$$\begin{array}{cccccccc} 1. & \text{abba} & : & \text{abab} & :: & \text{pqrrqp} & : & \text{?(pqrpqr)}\\ 2. & \text{abba} & : & \text{abbbbba} & :: & \text{pqrrpq} & : & \text{?(pqrrrrrpq)} \end{array}$$

A *signature* is a set *K* of *function symbols* where a nonnegative integer *n* is assigned to each *f* ∈ *K* making *f* an *n* *-ary function symbol*. A *K-algebra* is a nonempty set *C* where it is defined a family of finitary operations indexed by *K* i.e. to each *n*-ary function symbol *f* ∈ *K* corresponds exactly one *n*-ary operation *f*^*C*^ defined on *C*.

**Example 0.1.** *Let C be the set of all non-null strings on the letters a, b*, …., *z. Let C be endowed with two operations: “append” which is associated to the binary function symbol* λ, *and “symmetry” whose associated unary function symbol is σ. The “append” operation inputs two strings s*_1_ and *s*_2_ *and outputs the single string s*_1_ *s*_2_. *The “symmetry” operation inverts the order of letters in its input string i.e. σ*(*l*_1_ *l*_2_…*l*_*n*−1_ *l*_*n*_) = *l*_*n*_ *l*_*n*−1_…*l*_2_ *l*_1_. *This structured set C is a K-algebra when considering the signature K* = {λ, *σ*} □.

Now, let us consider a countable set *V* = {*x*_1_, *x*_2_, …} of variables and let us generate the set of *K*-terms in the standard recursive way:

- every variable is a *K*-term,
- if *f* is an *n*-ary function symbol and *t*_1_, *t*_2_, …, *t*_*n*_ are terms, then *f*(*t*_1_, *t*_2_, …, *t*_*n*_) is a *K*-term.

This recursive generation of terms has some well known consequences (see [50–52]): First, each *assignment* of variables *α*: *V* → *C* can be extended to every term *t* = *f*(*t*_1_, …*t*_*n*_) by the recursive definition *α*(*t*) = *f*^*C*^(*α*(*t*_1_), …., *α*(*t*_*n*_)). And second, if {*x*_1_, *x*_2_, …, *x*_*n*_} is the set of variables occurring in the term *t*, then an *n*-ary operation *f*_*t*_ is determined on *C* by *f*_*t*_(*a*_1_, *a*_2_, …, *a*_*n*_) = *α*(*t*) where the assignment *α* satisfies *a*_1_ = *α*(*x*_1_), …, *a*_*n*_ = *α*(*x*_*n*_). Notice that any nonempty set Π of *K*-terms determines a family of operations {*f*_*t*_}_*t* ∈ Π_ in the *K*-algebra *C*. The *closure* of *A*_0_ ⊆ *C* with respect to {*f*_*t*_}_*t* ∈ Π_ is the countable union ${⋃}_{i=0}^{\infty }A_{i}=A_{0}\cup A_{1}\cup A_{2}...$ where *A*_*i*_ = {*f*_*t*_(*a*_1_, *a*_2_, …, *a*_*n*_) | *t* ∈ Π and *a*_1_, *a*_2_, …, *a*_*n*_ ∈ *A*_*i*−1_}.

**Definition 0.1.** *Let C be a K-algebra, let A* ⊆ *C, and let* Π *be a set of K-terms. The pair (A,* Π) *is a domain with context C if the set A is closed with respect to the family of operations {f*_*t*_}_*t*∈Π_. *Also, we say that A*_0_ ⊆ *A generates* (*A*, Π) *if A is the closure of A*_0_ *with respect to* {*f*_*t*_}_*t*∈Π_.

**Example 0.2.** *Let us consider the K-algebra from example 0.1 and consider two K-terms: t* = λ(*x*_1_, *x*_2_) *and s* = λ(*x*_1_, *σ*(*x*_2_)). *Notice that f*_*t*_ *represents the append operation. In contrast, the operation f*_*s*_ *takes two strings and appends to the first one, the result of applying symmetry to the second one. Given* Π = {*t*, *s*}, *let us consider the domain* (*A*, Π) *generated by the two-string set A*_0_ = {*ab*, *p*}. *For illustrative purposes, let us emphasize that A is an infinite set containing elements like ab, p, abp = f*_*t*_(*ab*, *p*), *pba* = *f*_*s*_(*p*, *ab*), and *abpabp* = *f*_*t*_(*abp*, *abp*) = *f*_*s*_(*abp*, *pba*). □.

We say that *a* ∈ *A* is an *element* of (*A*, Π) and that *f*_*t*_ is an *operation* of (*A*, Π). In Example 0.2 the elements are strings of letters and the operations are syntactic operators. To introduce our formalism, let us first consider a similar, widely known algebraic notion. Let *A* and *B* be two *K*-algebras. A map *h*: *A* → *B* is called a *homomorphism* from *A* to *B* if for each *n*-ary function symbol *f* ∈ *K* and every *a*_1_, …, *a*_*n*_ ∈ *A*,

$$\begin{array}{c} h\left(f^{A}\left(a_{1},...,a_{n}\right)\right)=f^{B}\left(h\left(a_{1}\right),...,h\left(a_{n}\right)\right) \end{array}$$ (1)

This algebraic definition formalizes the preservation of the structure of the *K*-algebra *A* into the *K*-algebra *B*. As mentioned in the introduction, notions such as *quasi-homomorphisms* [4] and *local-homomorphisms* [8] have been proposed as models for analogy. A key problem with these approaches is that many mathematical constructions—such as products, quotients, limits, adjunctions and others whose construction depends on homomorphisms—lose their key properties when “exceptions” are allowed (the composition of two partial-functions or quasi-homomorphisms is not well defined in general, hence these notions may not satisfy the composition axiom required by category theory [9, pp. 7]).

To preserve the potential usefulness of these constructions in modeling of cognitive phenomena, we propose a model based on homomorphisms. But we need a strategy to achieve certain flexibility for formalizing at least some of the analogies with psychological interest. To this aim, we have included a set of parameters (the set Π of Definition 0.1) that gives us a margin of action in the modeling process. A first version of our model is presented now.

**Definition 0.2.** (RMA/Restricted Model for Analogies) *Let* (*A*, Π) *and* (*B*, Π) *be two domains whose context is the K-algebra C*. *A map h*: *A* → *B* *is called a homomorphism from* (*A*, Π) *to* (*B*, Π) *and denoted by h*: (*A*, Π) → (*B*, Π) *if for each t*(*x*_1_, *x*_2_, …, *x*_*n*_) ∈ Π *and every* *a*_1_, *a*_2_, …, *a*_*n*_ ∈ *A*,

$$\begin{array}{c} h\left(f_{t}^{A}\left(a_{1},...,a_{n}\right)\right)=f_{t}^{B}\left(h\left(a_{1}\right),...,h\left(a_{n}\right)\right) \end{array}$$ (2)

The only difference between conditions (1) and (2) is that the last condition ensures the preservation of the structure determined by the family of operations {*f*_*t*_}_*t* ∈ Π_ instead of the operations that are indexed by the signature *K*. This subtle characteristic together with the notion of “generating a domain” endow our model with certain flexibility when formalizing analogies as it is shown by the next examples.

**Example 0.3.** *Let us model the analogy problem abba*: *abab*::*pqrrqp*:? *and propose a solution through the model*.

*Let us consider the set of terms* Π = {*t*, *s*} *from Example 0.2 where the term* *t* = λ(*x*_1_, *x*_2_) *gives the operation “append” and s* = λ(*x*_1_, *σ*(*x*_2_)) *gives an operation which appends a first string to the result of applying symmetry to a second string. Let* (*A*, Π) *be the domain generated by the singleton A*_0_ = {*ab*} *and let* (*B*, Π) *be the domain generated by the singleton B*_0_ = {*pqr*}. *Observe that abba, abab* ∈ *A and that pqrrqp* ∈ *B*. *We need to look for a homomorphism h*: *A* → *B that extends the partial map h*′(*ab*) = *pqr*. *There exists a (unique) homomorphism h from the domain* (*A*, Π) *to the domain* (*B*, Π) *that extends h*′ (*see* S1 Note). *Since h is a homomorphism of domains, it must satisfy that h*(*abba*) = *pqrrqp and h*(*abab*) = *pqrpqr which is the answer that this particular modeling of the problem provides*. □

The example above formalizes a relation between the two key letter-strings in the source domain. This relation allows determining the missing item in the target domain via analogical transfer: *abba* and *abab* are built from the same element (*ab*) by applying the operations *f*_*t*_ and *f*_*s*_ imposed on the source domain (see S2 Note for more details about this relation and its transfer). Notice also that the definition of (*A*, Π) gives these two operations a key role, while downplaying the role of the original “symmetry” operation. These observations are consistent with empirical data suggesting that analogy is based on the mapping of relations [6, 53] and is able to emphasize certain features of domains while downplaying certain others [54].

Clearly, our definition of domains is reminiscent of similar proposals in the literature (see [5, 8, 40]) where a domain is a mathematical set together with a collection of operations defined on it. However, the syntactic parameters in the set Π, besides giving some flexibility to our model, gives us a way of “quantifying” the representational structure that a domain imposes over an analogy problem. To see this, observe that under the perspective of the *K*-algebra *C* (Example 0.1), the structure imposed on the string *abba* is “low” because this string can be built up in many ways from the alphabet and the primitives λ and *σ*. But under the perspective of (*A*, Π) in Example 0.3, the structure imposed on *abba* is “high” because there is exactly one way of building such a string: the only way in which the domain (*A*, Π) can “understand” the letter-string *abba* is *f*_*s*_(*ab*, *ab*). Next example displays another domain that “understands” the string *abba* in a unique way.

**Example 0.4.** *Let us model the proportional analogy*

$$\begin{array}{c} abba:abbbbba::pqrrpq:? \end{array}$$ (3)

*Let us consider the set* Π = {*t*_1_, *t*_2_} = {λ(λ(*x*_1_, *x*_2_), λ(*x*_2_, *x*_1_)), λ(λ(λ(λ(λ(*x*_1_, *x*_2_), *x*_2_), *x*_2_), *x*_2_), λ(*x*_2_, *x*_1_))} *and observe that* $f_{t_{1}}\left(a,b\right)=abba$. *At the same values, the operation determined by t*_2_ *returns the string abbbbba. Therefore, when considering* (*A*, Π) *as the domain generated by the set A*_0_ = {*a*, *b*} *we conclude that abba, abbbbba* ∈ *A*. *Also, if* (*B*, Π) *is the domain generated by the set B*_0_ = {*pq*, *r*}, *then* *pqrrpq* ∈ *B*. *We now look for a homomorphism h from* (*A*, Π) *to* (*B*, Π) *that extends the partial map given by h*′(*a*) = *pq* *and* *h*′(*b*) = *r*. *It can be shown (see* S3 Note) *that there exists a (unique) homomorphism h from the domain* (*A*, Π) *to the domain* (*B*, Π) *extending h*′. *This map must satisfy h*(*abba*) = *pqrrpq* *and h*(*abbbbba*) = *pqrrrrrpq*. *This last string is the answer given by this particular model of the problem*. □

The last two examples show the parameters available in our model of analogy: The source and the target of the analogy are modeled through domains (*A*, Π) and (*B*, Π) respectively. A partial map *h*′—considered as the “analogical map”—characterizes the relation between the source and the target. The model takes this information and provides us with the “analogical transfer” given by *h*: (*A*, Π) → (*B*, Π).

We have been assuming that the source and the target of an analogy share the same context (the same *K*-algebra). And that the set Π determines the family of operations in source and target i.e. the operations in both domains share the same “syntactic structure”. These assumptions are systematically violated by most analogies because, in general, the source and the target of an analogy differ radically. To account for this observation, we need a way to relate terms of the two domains—because each one may have its own language.

Let *K*_1_, *K*_2_ be two signatures, let Π be a set of *K*_1_-terms and let Ψ be a set of *K*_2_-terms. A map *F*: Π → Ψ is called a *term translation* if it preserves the variables. More precisely, for each *t* ∈ Π, the set of variables occurring in *t* is the exact same set of variables occurring in *F*(*t*).

**Definition 0.3** (MMA/Mathematical Model for Analogies) *Let C*_1_ *be a K*_1_-*algebra and let C*_2_ *be a K*_2_-*algebra. Let* (*A*, Π) *be a domain with context C*_1_, *let* (*B*, Ψ) *be a domain with context C*_2_ *and let F*: Π → Ψ *be a term translation. A map h*: *A* → *B* *is called a F -homomorphism from* (*A*, Π) *to* (*B*, Ψ) *and denoted by h*: (*A*, Π) → (*B*, Ψ) *if for each term t*(*x*_1_, *x*_2_, …, *x*_*n*_) ∈ Π *and every a*_1_, *a*_2_, …, *a*_*n*_ ∈ *A*,

$$\begin{array}{c} h\left(f_{t}^{A}\left(a_{1},...,a_{n}\right)\right)=f_{F(t)}^{B}\left(h\left(a_{1}\right),...,h\left(a_{n}\right)\right). \end{array}$$ (4)

This is our core definition, and we refer to it as the *mathematical model for analogy* (MMA). Notice that Definition 0.2 is just a particular case of the MMA with *C*_1_ = *C*_2_, Π = Ψ and *F* equal to the identity. This model has two main components, namely, the “structure-preserving” map *h*: *A* → *B* and the “symbol system” determined by the map *F*: Π → Ψ. These two components are in line with the explanation given by Dedre Gentner for the striking abilities of human cognition when she says “…analogical ability is the key factor in our prodigious capacity, and, … possession of a symbol system is crucial to the full expression of analogical ability” [55].

In this sense, the MMA abstracts a process whereby chunks of information (from context *C*_1_) are combined and coded in (*A*, Π) for representing the source domain, and concurrently, other chunks of information (from context *C*_2_) are combined and coded in (*B*, Ψ) for representing the target domain. These two processes are coordinated in a way that both domains end up sharing a common structure characterized by the map *F*: Π → Ψ so that the analogical transfer can be performed by the map *h*: *A* → *B*. Each domain has two dimensions. The symbolic dimension is conformed by sets of terms (Π and Ψ) that represent symbolic information and embedded grammars. And the semantic dimension is conformed by sets (*A* and *B*) that abstract possibly non-symbolic elements such as conceptual objects or spatial coordinates.

Some features of this definition can be illustrated through the example: “suppose the letter-string *aabc* were changed to *aabd*; how would you change the letter string *ijkk* in the same way?” [42]. The copycat model reports that the most preferred answer is the letter string *ijll* followed by *ijkl* and then by *hjkk*. The next example uses the MMA to provide us with insight about the algebraic nature of the solution *hjkk*.

**Example 0.5.** *Let us model the proportional analogy*

$$\begin{array}{c} aabc:aabd::ijkk:? \end{array}$$ (5)

*Let us endow the K-algebra C (Example 0.1) with the additional operation “successor” and its inverse “predecessor”, denoted by γ and γ*^−1^ *respectively. They are defined in a way that agrees with the alphabet ordering i.e. γ*(*a*) = *b*, *γ*(*b*) = *c*, …, *γ*(*y*) = *z*, *γ*(*z*) = *a*, *γ*^−1^(*z*) = *y*, *γ*^−1^(*y*) = *x*, *etc. Let F*: Π → Ψ *be the only term translation that can be defined between the following sets of terms*.

$$\begin{array}{c} \begin{array}{ccc} \Pi & = & \left\{\lambda \left(x_{1},x_{2}\right)\;,\;\;\gamma \left(x_{3}\right)\right\}\\ \Psi & = & \left\{\lambda \left(x_{2},x_{1}\right)\;,\gamma^{-1}\left(x_{3}\right)\right\} \end{array} \end{array}$$

*Now, let* (*A*, Π) *and* (*B*, Ψ) *be the two domains generated by the singletons A*_0_ = {*a*} *and B*_0_ = {*k*}, *respectively. Observe that aabc, aabd* ∈ *A, and that ijkk* ∈ *B*. *We look for an F-homomorphism h*: *A* → *B* *satisfying h*(*a*) = *k*. *It exists (see* S4 Note *) and can be defined recursively by h*(*γ*(*c*)) = *γ*^−1^(*h*(*c*)) *and h*(λ_*S*_(*c*_1_, *c*_2_)) = λ_*S*_(*h*(*c*_2_), *h*(*c*_1_)) *for every c, c*_1_, *c*_2_ ∈ *A*. *This model gives the intended answer of the analogy problem since h*(*aabc*) = *ijkk and h*(*aabd*) = *hjkk*. □

The example above illustrates how the complete model is more flexible than the restricted model. However, the constraints imposed by the MMA on the modeling of analogies are still strong. The nature of these constraints is illustrated in the next example where the model cannot give a solution.

**Example 0.6.** *Let us model the proportional analogy*

$$\begin{array}{c} ababa:abbaa::cdcdg:? \end{array}$$ (6)

*Let us set* Π = {λ(λ(*x*_1_, *x*_1_), *x*_2_), λ(λ(*x*_1_, *σ*(*x*_1_)), *x*_2_)}. *Let* (*A*, Π) *be the domain generated by the set A*_0_ = {*ab, a*} *and let* (*B*, Π) *be the domain generated by the set B*_0_ = {*cd*, *g*}. *Notice that ababa, abbaa* ∈ *A and cdcdg* ∈ *B*. *Now, let us notice that the MMA cannot provide an answer to this problem because an F-homomorphism h that extends h*′ *can not exist. Let us write* Π = {*t*_1_, *t*_2_} *and observe that* $f_{t_{1}}^{A}\left(ababa,a\right)=f_{t_{2}}^{A}\left(ababa,a\right)=ababaababaa$, *and then such an h would satisfy* $h\left(ababaababaa\right)=f_{t_{1}}^{B}\left(cdcdg,g\right)=cdcdgcdcdgg$ *and also* $h\left(ababaababaa\right)=f_{t_{2}}^{B}\left(cdcdg,g\right)=cdcdggdcdcg$. *This would imply that cdcdgcdcdgg = cdcdggdcdcg which is a contradiction. Therefore, h can not exist*. □

The lack of a solution is due to the fact that each binary operation *f*_*t*_ along with each pair of elements *x*, *y* ∈ *A* determine one constraint, namely

$$\begin{array}{c} h\left(f_{t}\left(x,y\right)\right)=f_{t}\left(h\left(x\right),h\left(y\right)\right) \end{array}$$

All these constraints must be satisfied to ensure that (*A*, Π) and (*B*, Ψ) share the same structure. The last example illustrates a case where one of these constraints is violated, meaning that the source and target domains have different structures. This suggests that if one does not plan using category theory to analyze phenomena, one might want to consider instead only a small number of these constraints—as in the definition of *local homomorphisms* [8, Definition 10].

All the examples until now suggest that the partial map *h*′ somehow forces the unique way in which *h* must be defined. This does not mean that the MMA predicts only one “valid” answer for each analogy problem. We can use the same framework, even the same K-algebra, and come up with a variety of different domains that yield different answers for the same problem. We illustrate this situation in the next example motivated by results given by Copycat: *kjh*, *kjj* and *lji* are solutions to the problem *abc*: *abd*::*kji*:?

**Example 0.7.** *Let us model the proportional analogy*

$$\begin{array}{c} abc:abd::kji:? \end{array}$$ (7)

*Let us consider the K-algebra C from the last example: the operation “successor” is denoted by γ and its inverse “predecessor” by γ*^−1^. *Now, let F*_1_: Π_1_ → Ψ_1_ *be the term translation between the sets of terms* Π_1_ = {λ(*x*_1_, *x*_2_), *γ*(*x*_3_)} *and* Ψ_1_ = {λ(*x*_1_, *x*_2_), *γ*^−1^(*x*_3_)}. *Also, let* (*A*, Π_1_) *and* (*B*, Ψ_1_) *be generated by the singletons A*_0_ = {*a*} *and B*_0_ = {*k*}, *respectively. Notice that abc, abd* ∈ *A and kji* ∈ *B*. *The F-homomorphism h*: *A* → *B satisfying h*(*a*) = *k* *exists. It can be defined recursively by h*(*γ*(*c*)) = *γ*^−1^(*h*(*c*)) *and h*(λ_*S*_(*c*_1_, *c*_2_)) = λ_*S*_(*h*(*c*_1_), *h*(*c*_2_)) *for every c, c*_1_, *c*_2_ ∈ *A*. *This first model entails h*(*abc*) = *kji and the answer h*(*abd*) = *kjh*.

*Now, let F*_2_: Π_2_ → Ψ_2_ *be the term translation between* Π_2_ = {λ(*x*_1_, *x*_2_), *γ*(*x*_3_)} *and* Ψ_2_ = {λ(*x*_2_, *x*_1_), *γ*(*x*_3_)}. *Also, let* (*A*, Π_2_) *and* (*B*, Ψ_2_) *be the two domains generated by the singletons A*_0_ = {*a*} *and B*_0_ = {*i*}, *respectively. Notice that abc, abd* ∈ *A, and that kji* ∈ *B*. *The technique used in Example 0.5 can be used to show that the F-homomorphism f*: *A* → *B satisfying f*(*a*) = *i* *exists. It can be defined recursively by f*(*γ*(*c*)) = *γ*(*f*(*c*)) *and f*(λ_*S*_(*c*_1_, *c*_2_)) = λ_*S*_(*f*(*c*_2_), *f*(*c*_1_)) *for every c, c*_1_, *c*_2_ ∈ *A*. *This second model entails f*(*abc*) = *kji* *and the answer f*(*abd*) = *lji*. □

In the next section we present three objects of study that illustrate how this model enables the application of abstract structures from category theory for studying analogy-related cognitive phenomena. But first, we need to present an adaptation of this model to build a representation of a symbolic model of analogy which will be used in our analysis of re-representation and flexibility.

### A formal model for symbolic models of analogy

According to literature, symbolic models are characterized by the following features [34, 56, 57]:

1. The use of formal languages to represent domains of knowledge as sets of terms and formulas.
2. The “analogical map” is obtained by the application of algorithms on symbolic descriptions. They implement a syntax-guided correspondence between representational elements from the source and the target.
3. The ultimate goal is to perform the “analogical transfer”. The source’s knowledge is projected into the target to hypothesize new statements about the target.

We want to emulate these features in a mathematical model that captures the behavior of symbolic models along with their key symbolic mechanisms to represent domains of knowledge. To this aim, let *X* = {*x*_1_, *x*_2_, …} be any set (whose elements are thought here as variables), let *K* be a signature, *t*, *t*_1_, *t*_2_, …., *t*_*n*_ be *K*-terms on *X*, and let us denote by $t\frac{t_{1},...,t_{n}}{x_{1},...,x_{n}}$ the “term” obtained by *substitution* i.e. simultaneously replacing each occurrence of *x*_*i*_ in *t* by the term *t*_*i*_. We can now define the set of terms Π*(*X*) that is “freely” generated by *X* (on a set of terms Π) as the minimal set that satisfies two conditions:

1. if *x* ∈ *X*, then *x* ∈ Π*, and
2. if *t* ∈ Π and *t*_1_, *t*_2_, …, *t*_*n*_ ∈ Π*, then $t\frac{t_{1},...,t_{n}}{x_{1},...,x_{n}}\in \Pi^{*}$.

Notice that each *K*-term *t* with variables *x*_1_, …, *x*_*n*_ determines a n-ary, symbolic operation *f*_*t*_ on this set Π*(*X*) as follows:

$$\begin{array}{c} f_{t}\left(t_{1},....,t_{n}\right)=t\frac{t_{1},...,t_{n}}{x_{1},...,x_{n}} \end{array}$$

Hence the pair (Π*(*X*), Π) is a domain, namely the *free* Π*-domain* on the set *X*. This allows us to define a symbolic version of a domain of knowledge. The following definition is a particular case of Definition 0.1.

**Definition 0.4** (Symbolic Domain). *Let K be a signature and let* Π *be a set of K-terms on the set of symbolic variables X. The pair* (Π*(*X*), Π) *is a symbolic domain with signature K. When the set X of symbolic variables is clear from the context we just call it a domain and denote it by* (Π*, Π).

We can think of the set Π* as the language that the symbolic model uses to describe a domain of knowledge. We regard the terms in Π and the operations in {*f*_*t*_}_*t*∈Π_ as the symbols and the rules (grammar) that determines such language. We now adapt the notion of a *F*-homomorphism with the aim to capture the behaviour of symbolic models of analogy and their aforementioned characteristics.

**Definition 0.5** (SMA/Symbolic Model for Analogy). *Let* Π *and* Ψ *be a set of K*_1_-*terms and K*_2_-*terms respectively. Let* (Π*, Π) *and* (Ψ*, Ψ) *be two symbolic domains and let F*: Π → Ψ be a term translation. A map F*: Π* → Ψ* *is called a term morphism when (1) it extends F and (2) it is a F-homomorphism from* (Π*, Π) *to* (Ψ*, Ψ).

The features (a), (b) and (c) of symbolic models of analogy are satisfied by a term morphism: The sets Π and Ψ are symbolic descriptions of the source and target that are provided as inputs to the symbolic analogy model. The term translation *F*: Π → Ψ associates source’s symbols with target’s symbols—it is a description of the characteristic “analogy map” that any symbolic model computes heuristically. The domains (Π*, Π) and (Ψ*, Ψ) are larger descriptions of the source and target created by the symbolic model by recursively using syntactic rules on the descriptions Π and Ψ. Finally, since F* translates the “larger description” Π* to the “larger description” Ψ* in a way that agrees with *F*, the notion of a term morphism formalizes the “analogical transfer” of knowledge that is based on the preservation of the syntactic structure of the representations of source and target.

The two last definitions comprise a formal model of computational symbolic models of analogy that captures a key mechanism implicit in the nature of symbolic models, namely that the analogical matching is guided by the syntax of the representational elements associated to the source and target domains. Clearly, computational symbolic models are more complex than our formal model and thus we do not expect a full description of them. Particular features, such as architectures or specific algorithms, are lost. Furthermore, our mathematical description does not accurately reflect a key feature of symbolic models: they represent knowledge by using higher-order formal languages whereas our formal description is restricted to using terms of first order languages. Still, the analyses performed on it provide (we believe) some conceptually interesting insights about computational symbolic models of analogy.

In order to analyze the role of re-representation in these computational models, we will explore a relation that appears between a “symbolic model” represented by a (term) morphism F*: (Π*, Π) → (Ψ*, Ψ) (Definition 0.5) and its underlying “conceptual analogy” represented by an *F*-homomorphism *h*: (*A*, Π) → (*B*, Ψ) (Definition 0.3). The core of this analysis is carried through the formal notion of a *coequalizer*—a generalization of a *quotient object*—that is introduced below in the context of category theory.

### Category theory

In category theory, the importance of diagrams and diagram chasing method has been emphasized as a notational method and as a device to organize ideas because “their use provides extensive savings in space and in mental effort” [58]. Our analysis of the cognitive design of board games in our first object of study is based on the use of diagrams that give a precise formal interpretation to the analogy between the spatial domain embedded in these board games and the numerical domain that is the mental representation to be learned. To this aim, we will need the notions of *object*, *morphism* and *commutative diagram* that are exemplified below. Although these notions are not directly defined in category theory, the examples below illustrate how these notions are instantiated in different applications of the theory.

#### Objects

As a way to introduce this notion, let us exemplify some objects: A *set* is a grouping of elements with no additional structure. A *group* (*G*, *) is a set *G* of elements together with an associative binary operation *, an identity element *e*, and for each *g* ∈ *G*, an inverse element *g*^−1^ ∈ *G* such that e*g = *g* = g*e and g*g^−1^ = *e* = *g*^−1^*g. We already introduced the notion of a *domain* (*A*, Π) that is a set *A* of elements together with a set Π of terms that determine a family of operations on *A*.

#### Morphisms

In category theory, a morphism *f*: *A* → *B* abstracts a particular relation between objects *A* and *B*. In our applications, the morphisms are structure preserving maps between objects. As examples, the morphisms between sets are mappings—no constraints on them. The morphisms between groups are group homomorphisms i.e. mappings *f*: (*G*, *e*, *) → (*H*, *e*′, •) that preserve identities (i.e. *f*(*e*) = *e*′) and satisfy *f*(*g*_1_*g_2_) = *f*(*g*_1_)•*f*(*g*_2_), for all *g*_1_, *g*_2_ ∈ *G*. The morphisms between domains (Definition 0.1) are pairs of maps (*F*, *f*) called *F*-homomorphisms (Definition 0.3). A morphism *f*: *A* → *B* is an *isomorphism* if there exists *g*: *B* → *A* such that *g* ∘ *f* = 1_*A*_ and *f* ∘ *g* = 1_*B*_, where 1_*A*_ and 1_*B*_ are the identity morphisms on *A* and *B*, respectively. If *g* exists, it is denoted by *f*^−1^ and called the *inverse* of *f*. An isomorphism conveys the idea that the two mapped objects are indistinguishable in terms of their structure. In mathematical contexts, the objects *A* and *B* in *f*: *A* → *B* are usually called the domain and the codomain of *f*, but such terminology is not adopted in this study because the word “domain” is used here to refer to a domain of knowledge in its psychological sense.

#### Category

Formally, a *category* *C* consists of a class of objects |*C*| = (*A*, *B*, …) such that for every pair (*A*, *B*) of objects, there is a set *C*(*A*, *B*) of morphisms from *A* to *B*. For every object *A*, there is an *identity* morphism 1_*A*_ ∈ *C*(*A*, *A*). There is also a composition operation “∘” that satisfies

- Identity: *f* ∘ 1_*A*_ = *f* = 1_*B*_ ∘ *f*, for all *f* ∈ *C*(*A*, *B*).
- Associativity: *h* ∘ (*g* ∘ *f*) = (*h* ∘ *g*) ∘ *f*, for all *f* ∈ *C*(*D*, *E*), *g* ∈ *C*(*E*, *F*) and *h* ∈ *C*(*F*, *G*).

We shall write *f*: *A* → *B* instead of *f* ∈ *C*(*A*, *B*) because, in our applications, morphisms are functions. The most familiar example of a category is **Set** where the objects are sets and the morphisms are functions; the identity morphism is the identity function and the operation is the usual composition of functions. Another well known example of a category is **Grp** whose objects are groups and whose morphisms are group homomorphisms. It is easily shown that domains (Definition 0.1) and pairs of mappings (*F*, *h*) (Definition 0.3) are respectively the objects and morphisms of a category that we denote here by **Dom**. When it does not lead into confusion, rather than making reference to the pair (*F*, *h*), we say that *h* is an *F*-homomorphism. Accordingly, if *F* is the identity term translation *I*, we say that *h* is an *I*-homomorphism.

#### Commutative diagrams

A diagram is a network or linear graph in which each vertex represents an object, and each oriented edge represents a morphism connecting the two objects at its ends. In a diagram, each path from an object *A* to an object *D* represents the morphism *f*: *A* → *D* that is obtained by composing all the arrows along the path. A *commutative diagram* is a diagram where each path from the same initial object to the same final object determines the same morphism (this does not apply to parallel arrows). In this manner, this kind of diagrams is useful to express a collection of simultaneous equations. For example, stating that Diagram 8 “commutes” means that the two composites *g* ∘ *f* and *v* ∘ *u* are equal. This condition is potentially satisfied by various collections of objects and arrows, for example, the sets and functions depicted in Diagram 9 below.

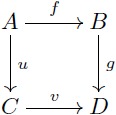
(8)

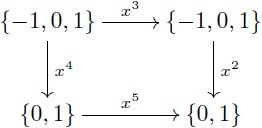
(9)

One goal of this study is to argue that the modeling of cognitive phenomena can be enriched by constructions provided by category theory. Such constructions are defined in terms of “universal mapping properties” (UMP) which provide a principled understanding of these constructions in terms of their relation to other objects in the same category. We will observe that when these abstract constructions serve as models for psychological phenomena, their universal mapping properties can be interpreted as features of such phenomena. Let us illustrate the notion of an UMP by considering the one that characterizes our free Π-Domain on a set *X*.

#### The UMP of a symbolic domain

A symbolic domain was defined as a free Π-domain on a set *X*. The UMP of this “free domain” is described as follows: There is the injection *i*: *X* → Π*[*X*], and given any domain (*A*, Π) and any assignment *α*′: *X* → *A*, then there is a *unique* *I*-homomorphism *α*: (Π*, Π) → (*A*, Π) that extends *α*′ i.e. such that *α* ∘ *i* = *α*′, all as indicated in the following diagram:

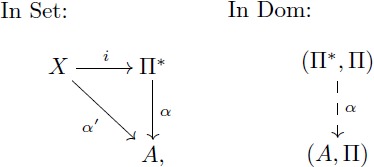


A key argument for the validity of symbolic models is that they are domain-general models of analogical processing i.e. they can be applied to a wide array of domains of knowledge. The UMP stated above may be taken as a formal characterization of such property: the symbolic model (Π*, Π) can “emulate” any semantic domain with form (*A*, Π) in a *unique* way through the mapping *α* (c.f. discussion in [56]). This UMP can be proved by standard algebraic procedures (for example by adapting the proof in [10]). And it permits the statement of the next lemma that is crucial to our applications. A proof of this lemma is given in supplementary text (see S1 Appendix).

**Lemma 0.1.** *Let F*: Π → Ψ *be a term translation, h*: (*A*, Π) → (*B*, Ψ) *be a F-homomorphism and α*′: *V* → *A be an assignment of variables. Let α* *and β be the extensions of α*′ *and h* ∘ *α*′ as in the diagram below. If there exists a term morphism F* *that extends F, then* diagram 10 *(below) commutes.*

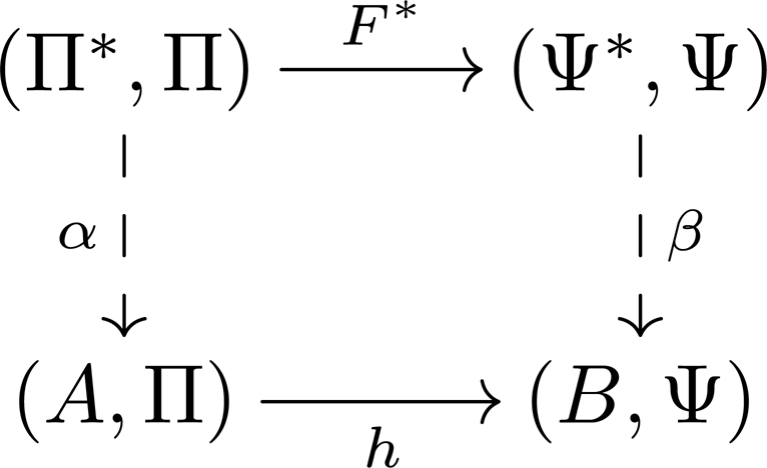
(10)

Now, let us consider another UMP, namely the one that characterizes a *quotient set* by an equivalence relation. A partition of a set *X* is a collection of disjoint subsets of *X* whose union is the whole set *X*. Such a partition determines a binary relation on *X* called an *equivalence relation* and denoted here by “∼”. The element of the partition that contains *x* is denoted by [*x*] and it is called the *equivalence class* of *x* because it contains all elements *y* ∈ *X* equivalent to *x*:

$$\begin{array}{c} \left[x]\;=\;\{y\in X\;|\;x∼y\right\} \end{array}$$

One can think of an equivalence relation as arising from the equivalent elements having some property in common (like being the same color). And one then regards the equivalence classes [*x*] as the properties themselves i.e. “abstract objects” (the colors: red, blue, etc.). The set of abstract objects (the set of colors) is thus defined as the set of equivalence classes which is known as the *quotient* of *X* by ∼ and determined as

$$\begin{array}{c} X/∼\;\;=\;\;\{\;\;[x]\;\;\;|\;x\in X\} \end{array}$$

This is known as “definition by abstraction”, and it describes, for example, the way that rational numbers are constructed from pairs of integer numbers. The quotient set is used to “abstract away” the difference between equivalent elements by identifying such elements. The *quotient map* *π*: *X* → *X*/∼ taking *x* to [*x*] has the property that every map *z*: *X* → *Z* respecting the equivalence relation (i.e. *x*_1_ ∼ *x*_2_ implies *f*(*x*_1_) = *f*(*x*_2_)) can be decomposed as *z* = *u* ∘ *π* where *u* is unique (see Diagram 11). These useful notions can be generalized and applied to other contexts through the UMP that characterizes a *coequalizer*.

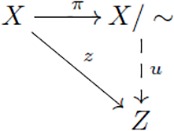
(11)

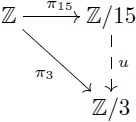
(12)

#### Coequalizer

Given a pair of parallel arrows *f*, *g*: *R* → *A* in a category **C**, a *coequalizer* of *f* and *g* consists of an object *Q* together with an arrow *π*: *A* → *Q* satisfying *π* ∘ *f* = *π* ∘ *g* and the following (universal mapping) property: For any object *Z* and any arrow *z*: *A* → *Z* satisfying *z* ∘ *f* = *z* ∘ *g*, there exists a *unique* arrow *u*: *Q* → *Z* such that *u* ∘ *π* = *z*. This UMP is depicted in commutative diagram 13.

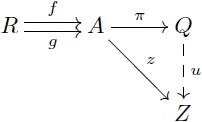
(13)

For example, in the category **Set** of sets, any equivalence relation *R* on a set *A*, provides two parallel arrows *r*_1_, *r*_2_: *R* → *A* which are the two projections of the inclusion *R* ⊆ *A* × *A*. Each (*a*_1_, *a*_2_)∈*R* forces *a*_1_ and *a*_2_ to be identified in the quotient set *Q* = *A*/*R*. The quotient map *π*: *A* → *A*/*R* defined by *a* → [*a*] is the coequalizer of *r*_1_ and *r*_2_ since it satisfies that for any *z*: *A* → *Z* that respects *R*, there is a unique *u* such that *u* ∘ *π* = *z*.

Another example can be given in the category **Grp** of groups. Let *N* ◁ *G* be any normal subgroup of *G* and form the semidirect product *G* × *N* having as elements the pairs <*x*, *n*> for *x* ∈ *G*, *n* ∈ *N* with the (associative) multiplication defined by <*x*, *n* > <*y*, *m*> = < *xy*, (*y*^−1^ *ny*)*m*>. In this case, the two parallel arrows *δ*_0_, *δ*_1_: *G* × *N* → *G* defined by *δ*_0_ < *x*, *n* > = *x* and *δ*_1_ < *x*, *n* > = *xn* have a coequalizer, namely the usual projection to the standard quotient group *π*: *G* → *G*/*N*. This notion of a quotient group will be further explored and exemplified below in the section “A First Isomorphism Theorem”.

In the category **Dom** of domains, any pair of parallel arrows (*F*, *f*), (*G*, *g*): (*R*, Ω) → (*A*, Π) determines a *congruence* on *A*, namely the least equivalence relation *R*′ on *A* that contains all pairs (*f*(*r*), *g*(*r*)). This determines the quotient set *A*/*R*′ which can be used to build the domain (*A*/*R*′, Π) whose operations are defined as *f*_*t*_([*a*_1_], [*a*_2_], …, [*a*_*n*_]) = [*f*_*t*_(*a*_1_, *a*_2_, …, *a*_*n*_)], for *t* ∈ Π. This domain is called the *quotient domain* of (*A*, Π) by *R*′ and denoted by (*A*, Π)/*R*′. The *I*-homomorphism defined by *a* → [*a*] is called the quotient map *π*: (*A*, Π) → (*A*, Π)/*R*′ and satisfies the UMP depicted in Diagram 13 above, which means that *π* is the coequalizer of *f* and *g*. Conversely, every *F*-homomorphism *α*: (*A*, Π) → (*Q*, Ψ) induces a congruence on *A* that determines a pair of *I*-homomorphisms *p*_0_, *p*_1_: (*K*, Π) → (*A*, Π) called its *kernel pair* and whose coequalizer is denoted by *π*_*α*_: (*A*, Π) → (*A*, Π)/*α*. These features of **Dom** imply a result that is relevant to our aims and that is generally know as a “first isomorphism theorem”.

#### A first isomorphism theorem

It is known that any category with kernel pairs and coequalizers admits a factorization system [59, proposition 4.2]. This means that each morphism in **Dom** can be factorized as a surjective *I*-homomorphism (the coequalizer of its kernel pair) composed with an injective *F*-homomorphism. A corollary of this result is that every surjective *I*-homomorphism *α*: (Π*, Π) → (*A*, Π) determines a quotient domain (Π*, Π)/*α* that is isomorphic to (*A*, Π). This kind of results is generally known as a “first isomorphism theorem” and provides us with a modeling tool since the quotient domain (Π*, Π)/*α* can be thought as a “symbolic representation” of the “semantic” domain (*A*, Π). This modeling tool will be developed further in our second object of study, but let us exemplify here the first isomorphism theorem without deepening into the details of the category **Dom**.

Let us provide an example in the more familiar category **Grp** which also has kernel pairs and coequalizers, and then admits an analogous factorization system. As a concrete example, the group of integers Z has identity 0, operation “addition” and for each integer *n*, its inverse is −*n*. For any fixed *m*, we can define [0]_*m*_ as the class of numbers whose remainders after division by *m* are zero. The quotient group Z/m is determined by considering the *m* classes given by [*k*] = {*h* + *k* | *h* ∼ 0} for *k* ∈ {0, 1, …, *m* − 1}. For instance, the quotient group Z/3 contains three elements: [0] = {…, −6, −3, 0, 3, 6, …}, [1] = {…, −5, −2, 1, 4, 7, …}, and [2] = {…, −4, −1, 2, 5, 8, …}. In this group, the identity is [0] and [1] is the inverse of [2]. Similarly, Z/15 can be thought as partitioning the integers into fifteen classes and then endowing these fifteen elements with a group structure. Here, the property of the quotient map *π*_15_ is that the associated equivalence relation is as small as possible, and thus one can always map Z/15 in a unique way to any other group that induces these identifications on Z by means of a morphism (e.g. Z/3 in Diagram 12).

For the case of groups, the first isomorphism theorem states that every surjective group homomorphism *α*: *G*_1_ → *G*_2_ determines an equivalence relation ∼ on *G*_1_ such that the quotient group *G*_1_/∼ is isomorphic to *G*_2_ (see Diagram 14). More precisely, the set of all classes [*x*] = {*y* ∈ *G*_1_ | *α*(*y*) = *α*(*x*)} can be endowed with a group structure that makes it indistinguishable from *G*_2_. For example, let *G*_2_ = {0, 1, 2; +} be the group with identity 0 and operation “addition modulo 3”. A surjective group homomorphism is given by the map $\alpha :Z\to G_{2}$ that assigns to *m* the remainder of the division of *m* by 3. The first isomorphism theorem tells us that *G*_2_ = {0, 1, 2; +} is isomorphic to Z/3 (see Diagram 15).

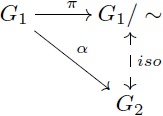
(14)

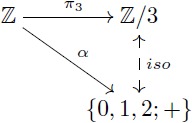
(15)

#### The theorem of re-representation

Our objects of study two and three are based on this theorem. We explain flexibility in symbolic models and build our formal models for the learning of fractions by using the commutativity of diagram 16 below. An intuitive interpretation of this theorem will be given during the analysis of re-representation and flexibility. The proof (see S1 Appendix) is direct from Lemma 0.1 and the first isomorphism theorem.

**Theorem 0.2** (Re-representation). *Let F*: Π → Ψ *be a term translation, h*: (*A*, Π) → (*B*, Ψ) *an F-homomorphism and α*′: *V* → *A an assignment of variables. Consider* diagram 16 where F* *is a term morphism that extends F, α is the extension of α*′ *and β is the extension of h* ∘ *α*′. *If α and β are surjective, then there exist domains* (Π*, Π)/*α and* (Ψ*, Ψ)/*β*, *I-isomorphisms a, b and surjective I-homomorphisms π*_*α*_ *and π*_*β*_ that make both triangles of the diagram commute. Also, h* = *b*^−1^ ∘ *h* ∘ *a is an F-homomorphism that makes* Diagram (16) *commute*.

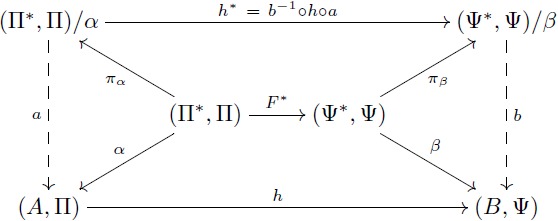
(16)

## Results

### Object of study 1

#### Board games and children’s learning of mathematics

We illustrate here how the MMA along with commutative diagrams can be used as a device to elaborate theories capable to describe empirical data. This object of study is based upon a series of experiments performed by Siegler and Ramani [32, 33] who showed that playing certain board games helps children to improve their mathematical knowledge. In two pretest-posttest experiments they assessed mathematical knowledge of children with four numerical tasks: (1) to *count* from 1 up to 10, (2) to *identify* the numerical phoneme associated to a numerical symbol, (3) to *compare* two numerals by pointing at the greater one, and (4) to *estimate* the position of a given number on a number line. The proficiency in these four tasks has been found predictive of the acquisition of further mathematical knowledge [60].

**Fig 3:**
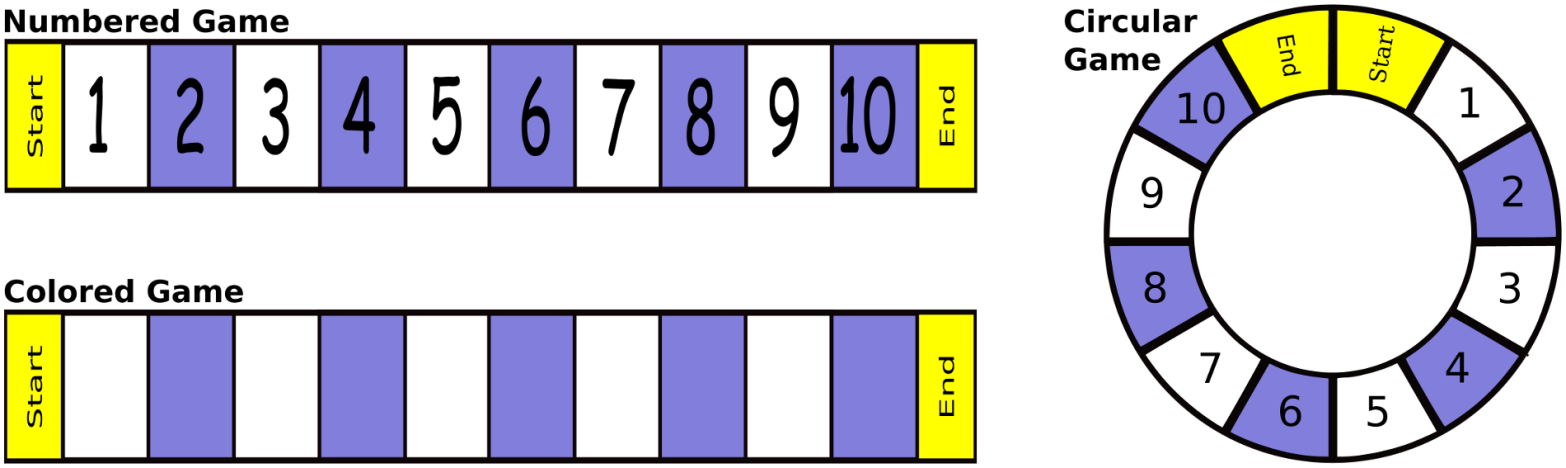
Each game has a different pattern of knowledge gains. (a) the colored game produced no gains of knowledge, (b) the circular game produced positive knowledge gains only in counting and identification, and (c) the numbered game produced knowledge gains in all four measures.

We conceptualize these two experiments as comparing the performance of children belonging to three playing groups. The three associated games (depicted in Fig 3) made each child race against an experimenter for completing a walk from “Start” to “End”. During this walk, the number of boxes advanced (1 or 2) in each round was determined by the flip of a coin. The game’s dynamics included saying each number (or color) at the time of stepping on it, and following certain protocol designed to control confounding variables.

The mentioned experiments were designed for testing the “representational mapping hypothesis” which states that the greater the transparency of the mapping between physical materials and desired internal representations, the greater the learning of the desired internal representations [32, 33]. The results showed that playing the “numbered game” significantly improves children’s learning of mathematics (see caption of Fig 3). The features of this game hypothesized to influence learning are (a) the linearity of the board, (b) the kinesthetic cues produced by physically moving the tokens, and (c) the game dynamics that help children identifying the value of the current number and counting-up from there. Also, the authors wonder whether the variety of redundant cues supporting the formation of linear representations of numerical magnitudes is crucial, as opposed to any particular cue being essential [32, 33].

These studies have shed a good deal of light on the nature of human learning. However, the authors acknowledge that some features of the intervention were based on hunches and guesses [61, pp. 393] which suggests that there is still room for new contributions to understand how the features of these games influence learning. Our goal here is to provide a more precise meaning for terms such as the “transparency of the mapping”, the “internal representations”, the “kinesthetic cues” or the “redundant cues”.

#### Analysis of board games and children’s learning of mathematics

In this section we use the MMA along with commutative diagrams to analyze the design of the educational games and, in the light of these analyses, to provide a complementary interpretation for the experimental results of Siegler and Ramani. The MMA will be used to represent a combination of semantic and syntactic cognition which has been argued to be characteristic of reasoning and acquisition of relational knowledge [62, 63]; in this sense, the MMA will play the role of a “painting set” enabling us to “draw and connect” the cognitive elements that shape the design of these games. Since the MMA by itself cannot explain the complex mechanisms underlying learning, we will need to decide among plausible mechanisms and competing hypothesis in order to theorize about the underlying phenomenon.

**Fig 4:**
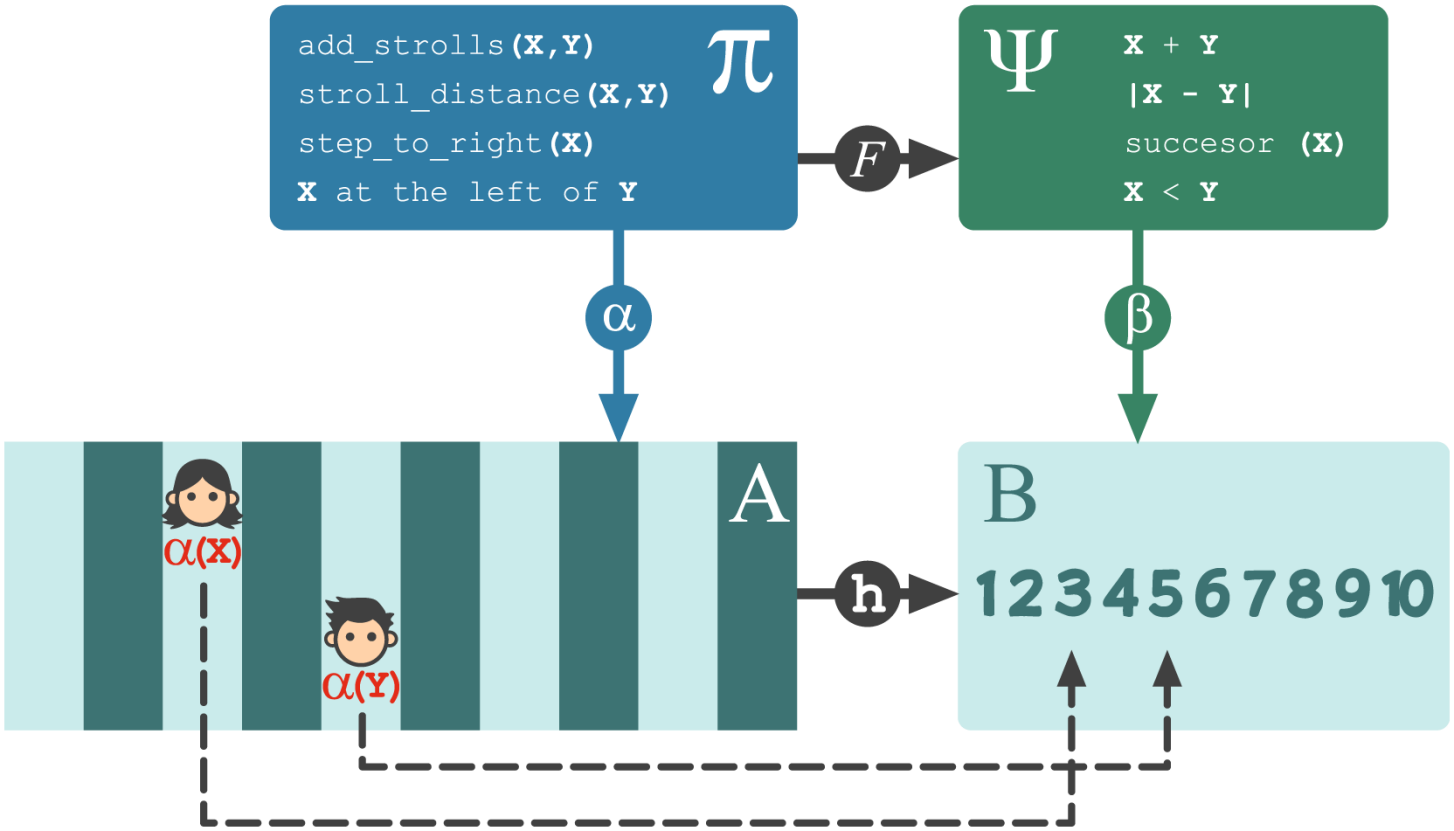
The numbered game (Siegler et al.) is represented as an *F*-homomorphism from a spatial source to a numerical target. Left side: a spatial domain formed by boxes and some spatial structure ordinarily well handled by children. Right side: a highly abstract domain of ten numbers. The map *h* is an *F*-homomorphism (Definition 0.3) that assigns the *i*-th number to the *i*-th box and preserves all the structure represented in the upper boxes.

Let *A* be a set of ten boxes spatially arranged and let *B* be the set of the first ten natural numbers. These two sets can be endowed with a certain structure as a way to model a spatial source and a numerical target in an analogy satisfying Definition 0.3. We have chosen the structure as indicated by Fig 4. The left half of the figure represents the source of the analogy: a concrete spatial domain that is familiar to children as they usually handle spatial notions. This domain is conformed by ten boxes (at the bottom) and some spatial relational knowledge (on top). Therein, a stroll that starts at the zero position and ends up at certain box is associated to the box itself, giving rise naturally to concepts such as the distance between two boxes. On the right half of Fig 4 is depicted the target domain—familiar to the game designer but unfamiliar to children: An abstract domain conformed by ten numbers (at the bottom) along with their usual arithmetical structure (on top). In describing this formal model, some technical issues must be addressed such as making a good definition for *successor*(10) or encoding the relationship *X* < *Y* in a way that satisfies the constraints imposed by the MMA. Since there are simple ways of handling these technical issues, and these are not crucial for this presentation, they are not addressed here.

The top half of Fig 4 depicts the syntactic component of the MMA: The symbolic representations of spatial relations (left side) are associated to the symbolic representations of numeric relations (right side) through the mapping *F*: Π → Ψ which acts as the identity on variables. This symbolic mapping assigns, for example, the numerical distance between two numbers to the stroll distance between boxes, and the successor of a number to the operation “go to right” in the game board. Similarly, the bottom half of the figure depicts the semantic (or conceptual) component of the MMA: Boxes in the spatial domain are associated to numbers in the numerical domain through the mapping *h*: *A* → *B* which assigns the number 1 to the first box from the left, the number 2 to the second box from the left, and so on.

The *interpretational mapping* *α* (see Fig 4) assigns semantic objects to syntactic symbols; it can be thought of as a dynamic binding that depends on the values taken by the symbol variables *X* and *Y*. For example, the two tokens depicted in the figure indicate that *α*(*X*) is the third box and *α*(*Y*) is the fifth box; and these two values fully determine the map *α*. For example, *α*(*go*_*to*_*right*(*X*)) is the fourth box. Also, by using the rule *β*(*X*) = *h*(*α*(*X*)) to assign *β*-values to variables, the interpretational mapping *β* is fully determined: *β*(*X*) is the number three, *β*(*Y*) is the number five, and the rest of values follow from there e.g. *β*(|*X* − *Y*|) is the number two.

In this context, the analogy can be characterized by the four mappings mentioned above. Fig 4 shows two ways in which a symbolic representation *s* of a spatial relation can be associated to a number: either (i) by following the horizontal arrow *F* and then the vertical arrow *β* or (ii) by following the vertical arrow *α* and then the horizontal arrow *h*. If for every possible mapping *α*, the routes (i) and (ii) are equivalent, then the pair of mappings (*F*, *h*) is an *F*-homomorphism. In particular, the diagram in Fig 4 commutes for each one of the 10^2^ possible mappings *α*, and then the mapping *h*: (*A*, Π) → (*B*, Ψ) is an *F*-homomorphism. This using of commutative diagrams can be seen as a formal statement of the *consistency requirement for analogical mapping* outlined by Holyoak and Thagard [5]. Moreover, this indicates that the MMA satisfies the notion of *consistency in a cognitive system* proposed by Halford and Wilson as a core characteristic of processing relational knowledge [3]. This point will be addressed in detail in the last section of the third object of study, but by now let us just highlight that the MMA integrates key properties of relational knowledge at the core of analogy-making.

Observe that *h* is an isomorphism, which means that the spatial domain (*A*, Π) and the numerical domain (*B*, Ψ) are indistinguishable in terms of their structure. This model thus provides an interpretation where the design of the numbered game entails a perfect analogy: the numbers printed on the boxes implement the isomorphism *h* that transfers the complete structure from the spatial domain to the numerical domain. Research in analogical transfer [64, 65] suggest that this game-play design provides cues on how to carry the relational structure from the spatial domain to the numerical representation. Hence, this perfect preservation of structure allows children to use *sound* working definitions that help them to acquire numerical relational knowledge such as the “successor of a number” which could be defined unconsciously as “advancing one box” during game playing.

Additionally, the two variables in the model (see *X* and *Y* in Fig 4) suggest that the using of *two tokens* is an important feature of the game: the racing dynamics engages the player’s attention on tracking and comparing the positions of the two competitors. Since each player is bonded to a variable whose value—the number printed on the box—changes with his position, the feeling of getting ahead or falling behind exemplifies the comparison of two numbers. A tie illustrates the equality of numbers. The number of single jumps necessary to go from one player’s location to the other’s indicates the distance between the two numbers being stepped on. Therefore, after a single match, the players have been exposed to dozens of good numerical examples involving two numbers. This analysis provides links with theories of cognitive development that describe relational complexity in terms of the number of symbols that must be cognitively processed [3, 13, 66]. And it suggests a prediction not accounted by Ramani and Siegler’s analysis: the numbered game would have less positive influence on learning if its design considered one token *only*, shared by the two players.

This pictures analogy-making in a way that is consistent with current theories of relational knowledge [63] and dual-processing accounts [62] which emphasize a combination of semantic and syntactic information for use in reasoning. For this study, we have conceptualized the “desired internal representation” as a collection of relation symbols which can be bounded to semantic elements (see the right side of Fig 4). Here, the “transparency of the mapping” can be assessed in terms of the relational knowledge that the pair of mappings (*F*, *h*) carry from the spatial to the numeric domain. These interpretations, along with studies showing that effective use of analogies promotes learning [44, 67–70], provide an explanation for the improvement of mathematical knowledge of children who played the numbered game. Actually, the analysis sketched above has been used in developing instructional materials that successfully promote children’s numerical knowledge [71]. This kind of research may open a path to investigate how relational representations can occur through structural alignment, symbol-binding and analogical mapping [72, cf.].

Additionally, the outlined analysis explains why the colored game produces no learning. The numbered game was represented through an *F*-homomorphism *h* defined from the “board without number labels” to the “desired representation of numbers”. Arguably, the colored game may be also represented by an F-homomorphism, but in this case, the target would not be a set of numbers. This suggests that the absence of numerical symbols hinders children from creating a representation of numbers that could be aligned with the spatial structure contained in the colored game (or aligning a numerical representation already existent).

Further elaborations with our model can also explain the effects on learning associated with the circular game. To this aim, let us clarify two reasonable assumptions that we have made regarding children’s perception of the games: (1) the distance between the two players is perceived visually as the length of the straight line connecting them, and (2) the spatial relation between the players is coded categorically: the first player can be at the “left/right” (or above/below) of the second player, even though they differ widely in their locations on vertical and horizontal axes.

Assumption (1) is supported by studies in space cognition that show that computing a route-distance following the circular path of the game would imply creating a “reference object” for each numbered box and then adding the different legs from one object to another along the route [73, 74]. This makes euclidean distance estimates more appealing because of cognitive economy. Additionally, Ramani and Siegler have explained that “such spatial cues are highly salient for young children, as indicated by their strong reliance on them on number conservation, liquid and solid quantity conservation…” [33, p. 547]. Likewise, assumption (2) is supported by empirical studies showing that categorical codings are used spontaneously [75].

The model and the analysis associated with the circular game can be built in a manner fairly similar to what was done for the numbered game. Actually, we imagine a model like that illustrated by Fig 4, but depicting a circular board instead of a linear one. The map *h* still transfers the notion of “successor” to the numerical domain but, due to the shape of the board, it cannot transfer the accurate distances nor the numerical comparisons: by assumption (1), the distance between the first and the tenth boxes is smaller than the distance between the first and the sixth boxes (Fig 3). By assumption (2), any criterion for comparing two boxes would make it impossible to order them in agreement with the order of numbers. For example, let us take the criterion left/right: it sets the first box to the left of the second box, and paradoxically, the tenth box to the left of these two (see Fig 3).

This analysis explains why the positive effect on learning produced by the circular game is weaker than the one produced by the numbered game. But it might be argued that children can perceive the circular path in ways that enable them to keep track of the actual numerical distances and comparisons. For example, by tracking the angular distance between the two tokens. However, such a notion of distance may not work for angles close to 360°, not to mention that it is still reasonable that assumptions (1) and (2) produce effects interfering with the correct coding of numerical representations.

To conclude, we should mention that our a priori knowledge of the experimental results may have influenced our modelling strategy. But the interpretation proposed here displays some appealing properties: (a) it provides new hypotheses and predictions—for instance, the “two token” hypothesis could be tested by an experiment wherein children in the experimental group would play a version of the numbered game whose design contemplated only one token meant to be shared by the two players. Of course, the challenge here would be to endow this new game with gaming dynamics that make it as fun as the original one; (b) it leads into a precise interpretation that is consistent with relational knowledge theory and experimental evidence: it gives further support to some of the conclusions reached by the original studies—for example, that the linearity of the game board is a key feature influencing learning; and (c) it associates specific features of the games with particular effects on the acquisition of numerical knowledge.

#### Relations versus associations

Relational knowledge is crucial to higher cognitive processes [13, 63, 66] and, as stated above, it plays a crucial role in the MMA. An elementary explanation based on associationist learning may posit that the acquisition of numbers is due to the learning of associations between representations of numbers (e.g. 1 → 2 → 3 → …) in which the stimulation of one representation activates the representation connected to it. In such an account, response times for certain inferences involving two numbers should depend on the distance between these numbers due to the required chain of activations. Notice however that an account based on relational knowledge with symbol-binding and structure-preserving maps such as the one depicted in Fig 4 would suggest parallel access to various representational items, only constrained by working memory capacities [66]. This makes a different claim because response times does not necessarily depend on the distance between the involved numbers. The two approaches thus suggest competing claims which can be assessed empirically.

Along these lines, the differences between the linear and circular boards can be further highlighted by drawing an analogy with research in transitive inference that makes a distinction between two tasks [13]: transitive inference and transitivity of choice. Transitive inference tasks typically present premises such as “Michael is taller than John”, “John is taller than Peter” and then ask for the taller person between Michael and Peter. In similar fashion, tasks of transitivity of choice are based on training subjects (such as monkeys) for example to choose one member of each pair in a series such as A+B-, B+C-, C+D-, D+E- (where “+” indicates a rewarded choice and “−” indicates nonrewarded choice). Subsequently, this kind of tasks asks subjects to choose one member of the pair BD (monkeys show a 90% of preference for B over D).

Though these two tasks are apparently similar, research has revealed deep differences among them including that transitivity of choice can be performed by very young children and a variety of species whereas transitive inference is limited to 4-year old (or older) humans. Current theories of relational complexity [13, 63, 66] account for these differences by pointing out that transitive inference tasks elicit ordering schemes that facilitate logically valid inferences whereas transitive of choice tasks elicit mechanisms that capture the results of training in the form of associations. Analogously, points scattered on a one-dimensional line can be (physically) well ordered which suggests that the linear board can elicit a structured schema. On the other hand, points scattered on a two-dimensional plane cannot be ordered following a natural criterion, and hence, by considering assumptions (1) and (2) of the presented analysis, the circular board cannot elicit a similar structured schema thus making it natural for associative mechanisms to be activated with consequences such as those mentioned above e.g. that the “distance association” between 1 and 10 would be stronger than the association between 1 and 6.

### Object of study 2

#### Re-representation and flexibility in symbolic models of analogy

Although the empirical evidence is neither systematic nor definitive, it points to the existence of mental processes that permit achieving certain flexibility in performing an analogical matching [49, 53]. However, leading accounts of analogy implement the principles of structural alignment [6] which seem to be insufficiently flexible to account for human analogical processing because non-structurally similar representational elements, although semantically compatible, are not permitted to match [76].

In order to account for this flexibility, computational models of analogy have implemented processes of re-representation: non-identical representational elements are considered matches and placed in structural correspondence during the analogical comparison [46, 53, 56]. The specifics of re-representation depend on the underlying theoretical framework, for example, the constraints of structure-mapping theory have been used to derive a theory where re-representation is a *selectively* activated process that identifies opportunities for re-representation when local changes can improve the overall match [77]. In contrast, the distributed representations of knowledge used by other approaches imply that analogy making is a persistent and naturally flexible process (e.g. LISA, BART and DORA models, [43, 45, 47, 48]). In the heuristic driven theory projection (HDTP) approach, re-representation is implemented through logical inference rules that operate as part of the matching process [78, see antiunification in]. Indurkhya’s interactionist approach argues that re-representation is the process by which a representational network is modified by grouping some of its elements with the aim to better reflect the structure of the target environment [40, p. 174].

Processes of re-representation are important for symbolic models of analogy which are particularly sensitive to the coding used for representing the source and target of an analogy. This sensitivity has often resulted in the use of hand-coded representations tailored to the needs of the model [34]. This limitation has been overcome by many symbolic models that have achieved certain flexibility in the processing of analogies. For example, PHINEAS—learning naive physics by analogical generalization—is able to take inputs created by qualitative simulations of physical behaviour [46]. More impressive is the case of a symbolic model that accesses open-source representation ontologies in order to solve physics problems by analogical model formulation [79].

The achievement of flexibility in some symbolic models has been attributed to the application of re-representation techniques: “our preferred technique for achieving flexibility … is to re-represent the nonmatching predicates into subpredicates …” [56, p.20]. Our aim in analyzing this object of study is to develop a formal explanation on how re-representation allows symbolic models to gain flexibility.

#### Analysis of re-representation and flexibility in symbolic models of analogy

As an aid to this presentation, let us illustrate the notion of re-representation by considering the following riddle: “In a room there are two fathers and two sons, but there are only three men. How is that possible?”. By definition, a symbolic model should represent this riddle by using a formal language whose signature may include symbols such as father, son, is_father_of, is_son_of, etc. Let us assume that this model uses the variables *F*_1_, *F*_2_ to represent the two fathers and *S*_1_, *S*_2_ for the two sons. To solve the riddle, the model has to “align” these four roles with only three persons (grandfather, father and son). This is usually done by means of an “inference calculus” composed by (a) some facts about the domain, and (b) some syntactic rules. These facts and rules may look as follows:

Fact 1) father(*S*_1_) = *F*_1_.
Fact 2) father(*S*_2_) = *F*_2_.
Rule 1) IF is_son_of(*X*, *Y*), THEN father(*X*) = *Y*.
Rule 2)father(son(*X*)) = *X*.

An inference calculus endows a symbolic model with abilities to process and discover knowledge. For our riddle, a chain of symbolic computations will be triggered to process the information which then results in the conclusion that one person is simultaneously being a father and a son, for example, that *F*_2_ = *S*_1_. This engenders re-representations because Fact 1 can be now re-represented as father(*F*_2_) = *F*_1_ and, by using Fact 2, another re-representation of Fact 1 is

$$\begin{array}{c} \text{father}\left(\text{father}\left(S_{2}\right)\right)=F_{1} \end{array}$$

The key observation here is that the latter statement is conceptually equivalent to Fact 1, but note also that these two statements are structurally different in regard to their syntax. And since symbolic models use the syntactic structure of representations as a guide to perform the analogical matching, the availability of multiple conceptually equivalent representations with different syntactic structures allows symbolic models to gain flexibility in the processing of the involved analogies.

Our goal here is to provide an alternative conceptualization of this process of re-representation mediated by an inference calculus. To this end, we outline its relation with a formal notion known as a quotient object. To this aim, we now use our definition of a symbolic domain to represent the riddle under analysis. Let us use the set of variables *V* = {*F*_1_, *F*_2_, *S*_1_, *S*_2_} and a set of terms Π that includes the terms in Table 1 above.

**Table 1:** Terms that generate the set Π*.

father(*X*)	is_father_of(*X*, *Y*)
son(*Y*)	is_son_of(*Y*, *X*)
TRUE	FALSE

The set Π* represents the language used by our symbolic model which contains an infinite number of terms. Examples of these terms are father(*S*_2_), is_father_of(*F*_2_, *S*_2_), is_father_of(father(*S*_2_), son(*F*_1_)), and father(son(*F*_2_)), among others. This set of terms can be seen as having certain structure: we can operate its elements through the syntactic operators *f*_*t*_ determined by the terms *t* ∈ Π. We have built the symbolic domain that is denoted by (Π*, Π).

We need to build another domain: a conceptual one that underlies the symbolic domain (Π*, Π). We use the domain (*A*, Π) whose elements are three persons (at least) and whose structure is given by the family relationships among them. For example, let us say, Angelo, Paul (Angelo’s father) and Marius (Paul’s father). Now, the solving of the riddle involves finding a *F*-homomorphim *α* that “aligns” the symbolic domain (Π*, Π) with the conceptual domain (*A*, Π). This can be done by assigning the conceptual objects to the symbolic variables in a way that induces the solution: Let us set *α*(*S*_2_) = Angelo, *α*(*F*_2_) = Paul, *α*(*S*_1_) = Paul and *α*(*F*_1_) = Marius. Notice that *α* can be extended to an *I*-homomorphism *α*: (Π*, Π) → (*A*, Π) in a *unique* way because of the UMP associated to (Π*, Π).

Building on these preliminaries, we can now explain how the notion of a coequalizer abstracts the idea of re-representation. The mapping *α* assigns objects to symbolic strings as illustrated in Table 2. This assignment determines the *quotient domain of* (Π*, Π) *with respect to* *α* denoted by (Π*, Π)/*α*. Each element of this quotient domain is a “class of terms” that acts as an “abstract representation” of some person in (*A*, Π). Crucially, the first isomorphism theorem ensures that (Π*, Π)/*α* is isomorphic to the conceptual domain (*A*, Π), and that the next diagram commutes.

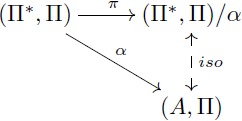
(17)

**Table 2:** Each term in the left column is associated to a conceptual element of (*A*, Π) in the right column. Each cell in the left column represents a class of the quotient domain (Π*, Π)/*α*.

*t* ∈ Π*	*α*(*t*)∈*A*
*F*_1_, father(*S*_1_), father(*F*_2_), father(father(*S*_2_)), …	Marius
*S*_1_, *F*_2_, son(*F*_1_), father(*S*_2_), …	Paul
*S*_2_, son(*F*_2_), son(*S*_1_), son(son(*F*_1_)), …	Angelo

Hence, a coequalizer abstracts the meaning of re-representation in this sense: each equivalence class contains all symbolic representations associated to one conceptual object, and since all these representations share the same meaning, all of them can be substituted between each other when representing knowledge associated to the domain (*A*, Π). This makes us think of *α* as an “interpretation map” that gives rise to the techniques of re-representation necessary to capture the semantics of the domain (*A*, Π). To put it another way, this mapping determines the re-representation techniques that must be implemented into a computational symbolic model to transform its language (Π*, Π) into a representation of (*A*, Π).

As we already mentioned, this interpretation of re-representation—characterized by the UMP of a coequalizer—sheds some light into an old discussion about why symbolic models of analogy have a capacity to simulate analogy in various contexts (general-domain) whereas computational models using conceptual “micro worlds” do not have such a capacity (specific-domain) [56, 80]. Additionally, we also mentioned that symbolic models generally use an “inference calculus” consisting on a set of facts and rules that (among other things) permits them to compute the aforementioned equivalence classes. This makes us interpret the quotient map *π*: (Π*, Π) → (Π*, Π)/*α* (in Diagram 17) as a representation of this “inference calculus” in the sense that it has a symbolic nature and contains enough information to identify all the representational elements in the language (Π*, Π) that are conceptually equivalent.

To make our proposal about how re-representation helps symbolic models to achieve flexibility, let us use the previously introduced riddle as a source analog for the almost identical riddle “In a room there are two mothers and two daughters, but there are only three women. How is that possible?”. The analogical alignment between the two riddles is self-evident, but the simplicity of this exercise will help us to shorten this presentation.

To formalize the new riddle, we use a different signature thus determining a symbolic domain (Ψ*, Ψ). As before, we model a conceptual domain (*B*, Ψ) to represent the three involved woman, let us say, Angela, Paula (Angela’s mother) and Mary (Paula’s mother). After relating these two domains through an *F*-homomorphims *β*, it turns out that the conceptual domain (*B*, Ψ) and the quotient domain (Ψ*, Ψ)/*β* are isomorphic. The equivalence classes of the quotient domain (Ψ*, Ψ)/*β* are illustrated in Table 3 below.

**Table 3:** Equivalence classes in the quotient domain (Ψ*, Ψ)/*β* associated to elements in the conceptual domain (*B*, Ψ).

*t* ∈ Ψ*	*β*(*t*)∈*B*
*F*_1_, mother(*S*_1_), mother(*F*_2_), mother(mother(*S*_2_)), …	Mary
*S*_1_, *F*_2_, daughter(*F*_1_), mother(*S*_2_), …	Paula
*S*_2_, daughter(*F*_2_), daughter(*S*_1_), daughter(daughter(*F*_1_)), …	Angela

Notice that the analogy between the two riddles can be seen now as operating at three levels. The diagram 16 in the model section depicts how, at a conceptual level, the mapping *h*: (*A*, Π) → (*B*, Ψ) preserves the conceptual structure by associating *Marius* with *Mary*, *Paul* with *Paula* and *Angelo* with *Angela*. It also depicts how, at a syntactic level, the mapping F*: (Π*, Π) → (Ψ*, Ψ) preserves the syntactic structure of symbolic representations. For example, to associate *Angelo* and *Angela*, this mapping would make the assignment son(*S*_1_) → daughter(*S*_1_), or the alternative

$$\begin{array}{c} \text{son}(\text{son}\left(F_{1}\right))\to \text{daughter}(\text{daughter}\left(F_{1}\right)). \end{array}$$

It is important noticing that the mapping F* *cannot* make the conceptually equivalent assignment

$$\begin{array}{c} \text{son}(S_{1})\to \text{daughter}(\text{daughter}\left(F_{1}\right)) \end{array}$$

because these two representations do not share the same syntactic structure. Hence, F* captures the behaviour of a symbolic model that has not yet achieved flexibility.

Our explanation of the arising of flexibility is based on an interpretation given to Diagram 16 (theorem of re-representation): On the one hand, the structure between conceptual domains is preserved by the mapping *h*: (*A*, Π) → (*B*, Ψ). On the other hand, the syntactic structure of symbolic descriptions associated to these domains is preserved by the mapping F*: (Π*, Π) → (Ψ*, Ψ). The mappings *α* and *β* associate symbolic descriptions to their respective conceptual domains. The first isomorphism theorem shows that the quotient domain (Π*, Π)/*α* is isomorphic to (*A*, Π) and that (Ψ*, Ψ)/*β* is isomorphic to (*B*, Ψ). The theorem proves the existence of an *F*-homomorphism h* between these two quotient domains. Although this mapping h* has a symbolic nature, it is indistinguishable from the conceptual mapping *h* because *a* and *b* are isomorphisms and h* = *b*^−1^ ∘ *h* ∘ *a*. Note that this mapping h*: (Π*, Π)/*α* → (Ψ*, Ψ)/*β* associates classes of equivalence and, in this sense, it may be seen as violating the preservation of syntactic structures because it *performs* assignments such as

$$\begin{array}{c} \left[\text{son}(S_{1})\right]\to [\text{daughter}\left(\text{daughter}\left(F_{1}\right))\right]. \end{array}$$

Hence, this mapping h* reflects the existence of *flexible* symbolic models of analogy: computational models that detect conceptually analogous representational elements and place them in correspondence, even though they do not share the same syntactic structure.

In sum, the theorem formalizes re-representation through a quotient domain; it describes flexibility as a byproduct of mapping equivalence classes that contain conceptually equivalent symbolic expressions that are dissimilar at a syntactic level. Therefore, this interpretation regards re-representation as an instance of a coequalizer that emerges in the context of symbolic models of analogy that achieve flexibility. In the next section we argue that this conceptualization is possible thanks to the MMA’s combining of syntactic and semantic information in its coding of knowledge. This point is developed by considering two approaches to re-representation that rely on purely symbolic methods.

#### Representing environments through symbols

The riddle-example used in the previous section can be approached through Unification Theory. This theory has a wide range of applications in the field of computational logic e.g. theorem provers, logic programming and equational theories. This theory helps in symbolic processing by providing tools to solve equations posed in sets of symbolic terms, for example, an equation between terms in our riddle-example could be posed as

$$\begin{array}{c} father\left(father\left(S_{2}\right)\right)=father\left(S_{1}\right) \end{array}$$

and a *solution* of such equation is a *substitution* *s*: *V* → *Term*[*V*] that “unifies” the two terms in the equation. In other words, when *s* = {*S*_2_ ← *S*_2_, *S*_1_ ← *father*(*S*_2_)} is applied to both terms (by replacing variables by terms), the terms become *syntactically* equal. Since unification theory has been given a category theory treatment in terms of co-equalizers [81], it may be tempting to think that this syntactic approach may be essentially the same as the one outlined above. We argue that the similarity is only superficial.

Substitutions are arrows *h*: *X* → *Y* in a category whose objects are sets. A substitution *q*: *X* → *Y* is said to *unify* a set of equations {*s*_*i*_ = *t*_*i*_: *i* ∈ *I*} in *X*, if for all *i* ∈ *I*, *q*(*s*_*i*_) = *q*(*t*_*i*_). Such unifiers do not always exist, but when they do exist, so does a *most general unifier (mgu)* defined to be a unifier *q*: *X* → *Y* such that for any unifier *q*′: *X* → *Y*′ there is a unique substitution *u*: *Y* → *Y*′ satisfying *u* ∘ *q* = *q*′. This definition of an mgu is transparently translated to the one of a coequalizer [81, p.175]. Let us now focus on the partition that is related to this coequalizer. This mgu induces a partition on the set of terms whose equivalence classes satisfy the following three properties [82, p. 19] (1) no equivalence class contains terms *f*(…) and *g*(…) with *f* ≠ *g*, (2) no term is equivalent to a proper subterm of itself, and (3) if [*f*(*s*1, *s*_2_, …, *s*_*n*_)] = [*f*(*t*_1_, *t*_2_, …, *t*_*n*_)], then [*s*_*i*_] = [*t*_*i*_] for *i* ∈ {1, …, *n*}. Let us notice that, in contrast with the equivalence classes depicted in Table 2, the property (1) implies that *father*(*S*_2_) and *son*(*F*_1_) cannot belong to the same class of equivalence i.e. these two terms cannot be conceptually equal under the Unification Theory approach described here. The difference outlined is due to the partition induced by an mgu is associated to a “syntactic equality” whereas the one induced by our approach is associated to a “conceptual equality” induced by the semantic component.

To further emphasize this contrast, we now refer to the approach to re-representation taken by the Structure Mapping Engine (SME) [7] which is a successful symbolic model of analogy. The earliest of its versions inputs source and target domains as descriptions written in a formal language (whose syntax determines the structure of the two representations). The SME processes the two domains by structurally aligning them in order to produce a set of correspondences between symbolic elements that characterize the analogical matching. To improve the performance of this analogical matching, recent versions of SME include re-representation modules that use techniques such as transformation, decomposition and entity spliting (among others) that permit substituting certain symbolic elements for others that are conceptually equivalent [77]. One transformation may replace the sentence greaterThan(sun, earth) by the conceptually equivalent lessThan(earth, sun). In this context, re-representation emerges as a a large collection of computational techniques designed to handle violations to the principles of structure-mapping theory [6].

Summarizing, the strength of the two symbolic approaches outlined above (Unification Theory and SME) is that they are theoretical approaches that count with an important body of computational support in the form of algorithms, libraries and computational modules ready-to-use in applications. The approach presented here complements them by providing a novel conceptualization of re-representation as a mechanism by which a “symbolic system” is able to create a coherent representation of an “environment”. This kind of conceptualization cannot be provided neither by Unification Theory nor by the SME approach. More importantly, this conceptualization is based on core principles of optimality, namely the UMP’s characterizing a free domain and a coequalizer. As Steven Phillips has pointed out, each UMP represents a kind of optimization of cognitive resources (see [19]). The UMP of a free domain can be interpreted here as a symbolic system being designed to represent a maximal variety of environments; and the UMP of a coequalizer can be associated to expending the minimal amount of cognitive resources for creating such representations.

These features also distinguishes the MMA from other purely algebraic accounts of analogical processing. For example, our approach might seem equivalent to Indurkhya’s interactionism [40] (see introduction) because both approaches depend on algebraic notions at their core. But the fact that the MMA can use the language of category theory gives it the ability to employ formal, principled constructions such as coequalizers and free objects in the development of cognitive models. This is significant because developing domain-general theories of analogical processing has been referred as crucial in advancing the understanding of analogy [56]. Additionally, the MMA is aimed to integrate syntactic and semantic information to afford representing relational knowledge whose importance has been highlighted by the accumulating evidence of the crucial role that relational knowledge plays in higher cognitive processes [63]. Hence, we believe this approach is not only useful for conceptualizing re-representation and symbolic models in an abstract manner, but also that it can shed some light on the functioning of certain human cognitive processes. This perspective is explored in our last object of study that develops a formal approach to the acquisition of the knowledge of fractions with basis on the theorem of re-representation.

### Object of study 3

#### Teaching and learning of fractions

The domain of rational numbers has traditionally been a difficult one for middle school students to master. Although most students eventually learn the specific algorithms that they are taught, their general conceptual knowledge often remains remarkably deficient. The foregoing errors are diverse and they all reveal a profound lack of conceptual understanding [83–86]. This calls our existing methods of teaching fractions into serious question. Hence, the development of theoretical tools to review teaching methods is necessary.

In this section we apply our theoretical approach to perform an analysis of the “area model” teaching strategy that is frequently used to teach fractions to children. This strategy assumes that students build their understanding of positive fractions as they partition wholes such as pizzas, brownies and other objects. The knowledge is thus transferred from a source domain with operations such as “put together” and “share between” by analogizing them with their corresponding arithmetic operations—addition and division—in the target domain of positive fractions.

Our formal interpretation of the teaching and learning of fractions is aimed to highlight relations between the notions of analogy, re-representation, conceptual knowledge and procedural knowledge. In this sense, we rely on observations by Dedre Gentner who claims that analogy, re-representation and learning are related: “a further way that learning can occur is re-representation: if there is good reason to believe two (nonidentical) relations should match (e.g., a very good overall structural match), then one or both of the nonmatching predicates may be rerepresented to permit the overall match” [55, p. 754].

Our goal here is to propose an abstract conceptualization of the learning of fractions that is based on the building of two formal models: The first one describes how a symbolic representation of the domain of fractions could be created and delivered by a hypothetical teacher. The second model describes some mechanisms whereby a hypothetical learner could acquire such symbolic representation. The analysis performed on these models provides a discussion on a long-standing and ongoing debate about the relations between two types of knowledge—conceptual and procedural [87].

#### Analysis of teaching and learning of fractions

We regard the area model teaching strategy as involving two conceptual domains, namely, the “pizza domain” and the “positive rational number line” which are modelled here through the domains (*A*, Π) and (*B*, Ψ), respectively (mathematically, each one of the sets *A* and *B* is the set $Q^{+}$ of positive rational numbers). These two conceptual domains are associated to two languages which are modelled here through the symbolic domains (Π*, Π) and (Ψ*, Ψ). They are generated up from the following sets of terms

$$\begin{array}{ll} \prod & =\{\text{put}\_\text{together}(X,Y),\text{ share}\_\text{between}(\text{X},\text{ Y}),\text{ smaller}\_\text{than}(\text{X},\text{Y})\}\\ \Psi & =\{\left(X+Y),\;(X/Y),\;(X<Y\right)\} \end{array}$$

**Fig 5:**
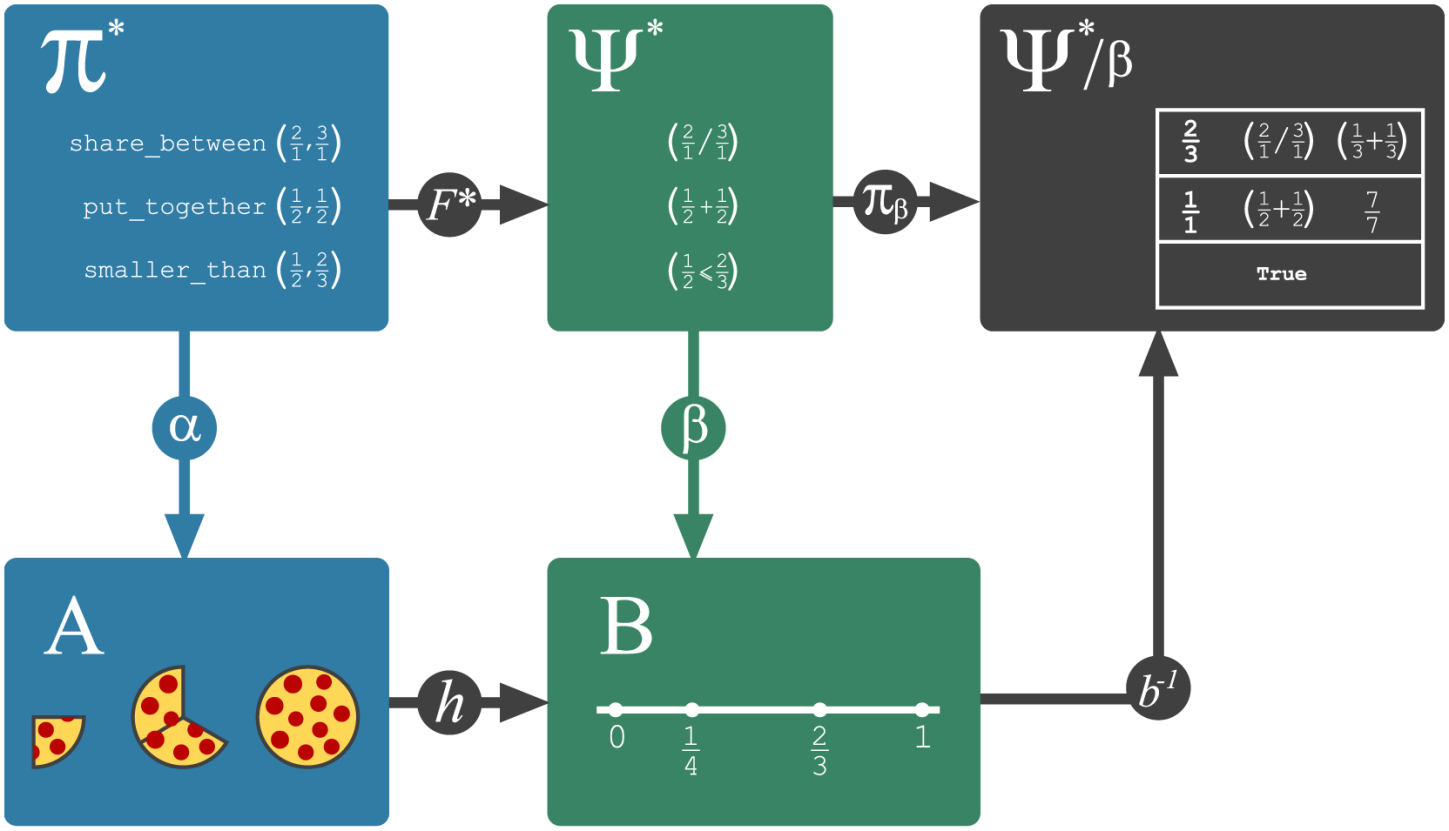
The *teacher’s model*. The domains (Ψ*, Ψ) and (*B*, Ψ) along with the map *β* allow the teacher to build the domain (Ψ*, Ψ)/*β* that represents the “positive rational number line”. This symbolic representation is delivered to learners by (1) handing the algorithms or procedural knowledge—map *π*_*β*_, (2) associating source and target domains through the language—map F*, and (3) interpreting conceptual knowledge—through a map *c*: *A* → Ψ*/*β* which coincides here with the composite *c* = *b*^−1^ ∘ *h*.

We can arrange the four aforementioned domains as in the bottom trapezoid of Diagram 16 (see Theorem 0.2). By the first isomorphism theorem, the quotient domain (Ψ*, Ψ)/*β* is isomorphic to the positive rational number line (see Fig 5). The theorem of re-representation ensures that the diagram depicted in Fig 5 commutes, which allows us to conceptualize such diagram as a model for the teaching strategy as we explain below. But first, we need to stress out that, in order to make this presentation more readable, Fig 5 refers to variables by abusing of the interpretation maps *α* and *β*: the figure depicts $V=\{\frac{m}{n}\;|\;m,n\in N,n\neq 0\}$ as the set of variables, but these variables are, a priori, just meaningless symbols of the language. Additionally, technical issues addressed in the construction of this model (the encoding of *X* ≤ *Y*, for example) are not detailed here due to space constraints.

The commutative diagram in Fig 5 is part of the diagram provided by the theorem of re-representation which is interpreted here as a model that describes our hypothetical teacher. This abstract model assumes, a priori, certain conceptual encoding of the positive rational number line i.e. the domain (*B*, Ψ). The model describes how this domain can be represented symbolically. To this aim, it assumes a capacity to process a standard grammar which permits generating the symbolic language for working with fractions, namely the domain (Ψ*, Ψ). The key point the model makes is that the interpretation *β* allows the transformation of this language into the domain (Ψ*, Ψ)/*β* which is isomorphic to the positive rational number line—by the first isomorphism theorem. This quotient domain is a symbolic representation of the rational numbers along with its structure (see Table 4). We will note below that this kind of symbolic representation can be acquired by a very simple cognitive agent as long as it counts with the capacity of processing a symbolic language and a set of specific rules to compute equivalence classes.

**Table 4:** Equivalence classes in the quotient domain (Ψ*, Ψ)/*β* associated to conceptual numbers in the domain (*B*, Ψ).

Equivalence classes in (Ψ*, Ψ)/*β*	Concepts in *B*
$\frac{1}{4}$, $\frac{3}{12}$, $\left(\frac{3}{24}+\frac{1}{8}\right)$, $\left(\frac{3}{36}+\frac{2}{12}\right)$, $\left(\left(\frac{2}{32}+\frac{2}{32}\right)+\frac{1}{8}\right)$, … $\left(\frac{1}{2}/\frac{2}{1}\right)$, $\left(\frac{1}{2}/\frac{6}{3}\right)$, $\left(\left(\frac{6}{3}/\frac{4}{1}\right)/\left(\frac{10}{1}/\frac{5}{1}\right)\right)$, …	a “quarter”
$\frac{1}{2}$, $\frac{6}{12}$, $\left(\frac{6}{24}+\frac{2}{8}\right)$, $\left(\frac{6}{36}+\frac{4}{12}\right)$, $\left(\left(\frac{4}{32}+\frac{4}{32}\right)+\frac{2}{8}\right)$, … $\left(\frac{1}{4}/\frac{1}{2}\right)$, $\left(\frac{1}{1}/\frac{6}{3}\right)$, $\left(\left(\frac{6}{3}/\frac{4}{1}\right)/\left(\frac{5}{1}/\frac{10}{2}\right)\right)$, …	a “half”
… …	… …
$\frac{1}{1}$, $\frac{12}{12}$, $\left(\frac{6}{24}+\frac{6}{8}\right)$, $\left(\frac{6}{36}+\frac{5}{6}\right)$, $\left(\frac{4}{32}+\left(\frac{4}{32}+\frac{3}{4}\right)\right)$, … $\left(\frac{1}{4}/\frac{1}{4}\right)$, $\left(\frac{5}{5}/\frac{3}{3}\right)$, $\left(\left(\frac{6}{3}/\frac{4}{1}\right)/\left(\frac{5}{1}/\frac{10}{1}\right)\right)$, …	“one”

The *learner’s model* is our second formal model. It describes how an “abstract learner” could acquire the symbolic representation given by the quotient domain (Ψ*, Ψ)/*β*. This model is depicted in Diagram 18 below and it uses the same elements depicted in Fig 5 but excludes the domain (*B*, Ψ) and the map *β*. This model is easier to interpret by using the inverse of F* which is denoted here by $F^{<\;*}$. As before, it is assumed that the learner has some capacity to process a grammar which permits generating the language (Ψ*, Ψ). Here, the “learning of the subject” occurs when our abstract learner is able to transform (Ψ*, Ψ) into the quotient domain (Ψ*, Ψ)/*β*.

The transformation mentioned above is achieved through the two arrows (Ψ*, Ψ) → (Ψ*, Ψ)/*β* depicted in Diagram 18: The mapping $\alpha \circ F^{<\;*}$ abstracts the process whereby the symbolic string $\frac{1}{2}+\frac{1}{2}$ is interpreted as two halves of a pizza that are grouped together in (*A*, Π), thus obtaining one pizza which afterwards is linked to the symbol 1 through the mapping *c* = *b*^−1^ ∘ *h*. The composite $c\circ \alpha \circ F^{<\;*}$ represents *conceptual knowledge*. Similarly, the map *π*_*β*_ abstracts the application of standard algorithms which show, for example, that the symbolic expression $\left(\frac{1}{4}\;/\;\left(\frac{3}{15}+\frac{3}{5}\right)\right)$ belongs to the same equivalence class that the symbol $\frac{5}{16}$. This quotient map *π*_*β*_ represents *procedural knowledge*. The two aforementioned kinds of knowledge are the center of a long-standing and ongoing debate in the learning of fractions [87].

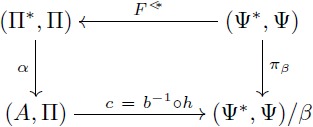
(18)

The *learner’s model*: The learner first generates the symbolic domain (Ψ*, Ψ) for then computing the equivalence classes that transform it into a representation of the “positive rational number line” (the quotient domain (Ψ*, Ψ)/*β*). To this aim, the learner can use the procedural knowledge (the map *π*_*β*_) or the conceptual knowledge (the composite $c\circ \alpha \circ F^{<\;*}$).

It is important noticing that diagram 18 commutes because it means that there is no discrepancy in using either of the two aforementioned mechanisms, that is, for every symbol *s* ∈ Ψ*, it holds that

$$\begin{array}{c} \pi_{\beta }\left(s\right)=c\circ \alpha \circ F^{<\;*}\left(s\right) \end{array}$$ (19)

which may reflect the kind of interactions observed in laboratory experiments, namely, that the procedural skill (*π*_*β*_) and the conceptual understanding ($c\circ \alpha \circ F^{<\;*}$) are deeply intertwined into the learning of fractions [88].

Along these lines, the learner’s model provides an account of certain relations between conceptual and procedural knowledge. In the first place, the equality Eq (19) indicates that both mechanisms potentially perform the same function [87]. However, the specificities of the involved mappings suggest that these two mechanisms are implemented in very different ways. Notice that *π*_*β*_ is a mapping between symbolic representations which suggests that procedural knowledge can be automatized thus allowing people to solve problems in a quick and effective way by using few cognitive resources [89]. In contrast, conceptual knowledge is more demanding as it is described by a composite of various mappings, some of which involve semantic domains. It requires relational representations of these domains [89] and the ability to coherently map symbols and semantics [63]. The above considerations suggest that conceptual knowledge is more flexible than procedural knowledge but less efficient [90].

Additionally, the UMP of the coequalizer *π*_*β*_ can provide a simple but formal description of errors in the learning of fractions. An error such as $\frac{1}{2}+\frac{1}{2}=\frac{2}{4}$, which is commonly made by learners [91], can be seen as a mapping *e* from the symbolic domain (Ψ*, Ψ) to certain representation (*Q*, Ψ) that is entailed by their mistaken procedures and that confounds the numbers “one” and “a half”. The UMP of *π*_*β*_ ensures that this mapping can be factorized as *e* = *w* ∘ *π*_*β*_ where *w* is unique. Hence, the map *w* characterizes the error in terms of the equivalence classes that are unduly identified. In this case, *w* identifies the classes of equivalence [1] and $\left[\frac{1}{2}\right]$.

In the view outlined above, conceptual and procedural knowledge are seen as “re-representation techniques” that transform the language (Ψ*, Ψ) into an isomorphic representation of the positive rational number line. Hence, the interpretation given here poses re-representation as a core process in the acquisition of the knowledge of fractions. This formal analysis shows, at least as a proof of concept, that the human capacities of re-representation, language processing and analogy making can set a mechanism whereby rational number meanings could be acquired and developed. Thus, the models developed here are consistent with claims that the human capacities for number rely on recursive computations and symbolic processing [55, 92–94].

The potential of these two models as a research tool may be illustrated by using them to describe the kind of “rote learners” that would often “perform calculations without knowing why”. To this aim, let us remove the maps $\alpha \circ F^{<\;*}$ and *c* = *b*^−1^ ∘ *h* from the learner’s model. Mathematically, the map *π*_*β*_ is enough by itself to determine all the adequate equivalence classes on Ψ* so that (Ψ*, Ψ)/*β* remains isomorphic to (*B*, Ψ). This mathematical fact reflects two well established facts in the literature of mathematics education. First, that many learners can excel at performing symbolic calculations even when they do not assign any meaning to such computations. And second, that this kind of rote learners cannot apply their syntactic knowledge to solve problems in realistic contexts possibly due to they cannot associate symbolic and conceptual domains [91, 95].

Additionally, these kind of analysis may indicate that rote learners lag behind those learners described by the learner’s model (diagram 18) possibly because, to solve a problem such as $\frac{2}{3}+\frac{1}{4}$, rote learners can only apply algorithms, whereas the others can also “intuit” that the solution is “close to” 1: they can imagine the solution as an area that almost fills one pizza. Consequently, these learners can quickly notice mistakes in their application of the algorithms by checking that the output is “close to” 1. This brief analysis suggests that the lack of a domain like (*A*, Π) may prevent rote learners from obtaining conceptual feedback such as the one exemplified above in terms of “closeness”.

The analysis also outlines how conceptual voids hinder the development of intuitions about fractions. In the case of the area model teaching strategy, these intuitions are grounded on geometrical notions such as “smaller areas”, “similar areas”, “equal areas” and others like these. Hence, teaching strategies that deliver conceptual domains successfully—perhaps through visual cues, comprehensive analogies, or well chosen examples—enrich learner’s knowledge with additional structures such as spatial references or geometrical notions [70]. Yet recent studies have found that the knowledge of fractions is better delivered by using number lines instead of object’s areas during learning [83–86]. This suggest that there is still room for contributions, and thus, the sort of analyses presented here could be applied with the aim to design novel and effective teaching strategies.

Additional work may reveal the scope of the approach introduced here. It served us here to build a novel conceptualization of the learning and teaching of fractions where the processing of a grammar and the computing of equivalence classes are the basis to create a representation for rational numbers. It also enabled us to formally describe the relations among analogy, re-representation, conceptual knowledge and procedural knowledge. And the analysis on rote learning makes us to believe that this approach could be applied to perform theoretical analyses on teaching strategies with the aim to predict their effects on learning.

In sum, the mathematical modeling presented here uses notions from category theory to describe some of the components involved in the learning of fractions while outlining their relations. Since our interpretation is compatible with empirical observations, we believe these models may provide a formal framework wherein observations could be placed to study their relation with the larger context, thus deepening our understanding of their meaning (see other frameworks in [83, 91, 93]). Perhaps future research projects indicate the extent to which these models can be applied to describe, explain or predict phenomena regarding the teaching and the learning of fractions. In this work, we only illustrate how the MMA along with the language of category theory can help us to create formulations for cognitive phenomena that are related to analogy.

### A distinction between syntax and semantics

The distinction between the syntactic and semantic versions of the MMA was leveraged in the last section to provide an account of procedural and conceptual knowledge. This distinction is similar to the one proposed by Halford and Wilson who employed category theory to develop a theory of cognitive development [3]. They pointed out that representations in thought must be general so that they can be transferred to situations not previously experienced and argued that representations in the form of relational knowledge are necessary. They described how symbol systems and environmental elements must be set in structural correspondence by building a formal model: a *cognitive system* is defined as a symbol system (a n-ary operation *f* defined on a set of symbols *S*), an environment system (a n-ary operation *g* defined on a set of environmental elements *E*) together with a mapping *a*: *S* → *E* that makes the next diagram commute:

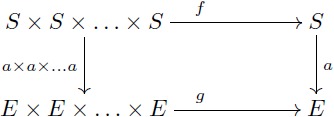
(20)

A *cognitive system*: the mapping *a*: *S* → *E* assigns symbolic representations to environment elements. The n-ary symbolic process *f* transforms a n-tuple of symbols into another symbol. The n-ary environmental process *g* transforms a n-tuple of semantic elements into another element. The commutativity of the diagram means that symbolic processes exactly reflect the corresponding processes in the environment system.

This definition allowed its authors to predict age-related differences in performance of certain cognitive tasks. This prediction was based on that the greater the number of symbols involved in a cognitive process (i.e. the arity associated to *f* or *g*), the greater the cognitive demands imposed on short term memory. These kind of thinking guided Halford, Wilson and Phillips to create an account of cognitive processing capacity in terms of the relational complexity of symbolic representations [66]. This is pointed out here because of the close relation between the MMA and a cognitive system: the mapping *a* in Diagram 20 is an *F*-homomorphism

$$\begin{array}{c} a:\left(S,\left\{f\left(x_{1},\ldots ,x_{n}\right)\right\}\right)\to \left(E,\left\{g\left(x_{1},\ldots ,x_{n}\right)\right\}\right). \end{array}$$

This may open a path to research how the relational complexity of representations influences analogical processing: the MMA extends the notion of a cognitive system to include the case of analogical processing where two symbol systems are interacting. In particular, Lemma 0.1 contributes by providing two “orthogonal” structure-preserving mappings: (1) between syntax and semantics within a domain of interest, and (2) between domains of interest (see Diagram 10).

It is important to point out here that we do not claim that symbolically structured cognition necessarily requires symbolic languages as part of their representational systems. This point is made by a model of analogy and schema induction (DORA) [48] which represents relations through four layers of units in a neural network. Such relations are composed by a relation symbol linked to roles which are bounded to fillers. Units representing a role oscillate in close temporal proximity with units representing the filler bound to that role, and out of phase with units representing other roles and fillers. The relational instance less_than(two, three) would be represented by units representing the first-object role of less_than oscillating in synchrony with units representing the number two, while units representing the second-object role of less_than oscillating in synchrony with units representing the number three. Units representing the first-object oscillate out of synchrony with units representing the second-object. Structure-consistent mapping occurs by concurrent activation of units in two analogues i.e. superimposing another relational instance (e.g. smaller_than) by having corresponding roles of all relational instances oscillate in synchrony.

In our perspective, the importance of these achievements lies partly in showing that the integration of semantic and syntactic information must not necessarily rely on the grammar of a symbolic language but rather it may be based on mechanisms similar to neural nets with synchronous oscillation. More importantly, DORA provides a precise account of how children transition from representing the world in terms of unstructured representations of objects to representing the world in a structured fashion where relational knowledge and structure-preserving mappings are explicit. The work presented here pushes a bit further these achievements by showing how similar premises can also provide a basis for the emergence of more complex structures such as those of procedural and conceptual knowledge in the learning of fractions. Although our models cannot give a precise account of the training process (in the way that DORA does), they at least suggest the core data structures and algorithms that may be used in a computational implementation aimed to simulate the human learning of fractions (similar to what is done in [94] where number meanings and other related concepts are learned from naturalistic data).

## Discussion

Category Theory can help us to understand how the core constructions of mathematics are systematically related to one another and how they arise from one another according to simple and general basic principles. By bridging a gap between the formal notions of category theory and the psychological notions of cognition, the MMA helped us to exploit these principles for thinking about the role of analogy in cognition: We used commutative diagrams to describe the learning by analogy that underlies the playing of board games. Also, we used a free domain and a coequalizer to explain the arising of flexibility in symbolic models of analogy. And we built formal models that suggest that the human abilities of re-representation, symbolic language processing and analogy making can explain the acquisition of knowledge of rational numbers. The coherence between the theoretical results and the empirical observations in literature supports that the approach presented here serves as a framework for modeling and analyzing cognitive phenomena related to analogy.

The approach seems to have at least two limitations. The first one is that the MMA imposes conditions that may be too strong for capturing analogical behaviour in certain contexts. But we have presented here three objects of study showing the existence of psychologically interesting phenomena that are well suited to be studied within this framework. The second limitation is that the MMA has less expressive power than computational frameworks of analogy that represent knowledge by using higher order languages. Although this comparison might be unfair because computational models pursue goals conceptually different from the ones pursued in this study, we acknowledge that the presented framework could be improved with the addition of higher order logics. In the meanwhile, this lack of expressiveness may well be compensated by the variety of formal notions (such as limits, colimits, adjuntions, functors and others) from category theory that can be used as construction blocks into the building of new cognitive models.

Even though the analyses presented in this work are based on simple models of complex phenomena, these models allowed us to articulate analogy-related cognitive theories and to exploit them into analyzing, explaining and organizing cognitive material. Hence, category theory not only helped us to re-conceptualize cognitive notions, but also to hypothesize on how these notions are connected to each other. This interplay between mathematics and cognitive theories yielded results that are mathematically interesting, conceptually revealing and potentially useful for the cases of re-representation and acquisition of numerical knowledge.

But, what other cognitive phenomena can be studied in this manner? We believe the approach presented here might help us to inquire into more fundamental aspects of analogy. For example, a problem of central concern to analogy researchers is to understand why a particular analogy is chosen over the possibly many other alternatives. It might be interesting to apply the MMA along with a theory of relational complexity and cognitive processing [66] in order to investigate whether the relational complexity of the analogical candidates can determine (or influence) the final selection. We suggest to move forward by using formal notions such as products, functors, limits and colimits into exploring more cognitive phenomena. Non-trivial results of category theory might become relevant in future research. For example, the free domain (Π*, Π) presented in this study is associated to a free functor that appears as the left adjoint to a forgetful functor defined on certain sub-categories of **Dom**. Hence, adjoint functor theorems might turn out to be suitable tools when studying the category of domains introduced here. We expect the full development of this framework will provide a large collection of mathematical tools to formulate theories that exploit the advantages of formal analysis in the study of the human cognitive architecture.

## Supporting information

S1 Note Proof for the existence of the homomorphism described in Example 0.3. (PDF)

S2 Note An alternative formalization of the relation between the two key letter-strings in the source domain of Example 0.3. (PDF)

S3 Note Proof for the existence of the homomorphism described in Example 0.4. (PDF)

S4 Note Proof for the existence of the homomorphism described in Example 0.5. (PDF)

S1 Appendix Proof for the rheorem of re-representation. (PDF)
