# Supplementary material for: Towards a category theory approach to analogy: Analyzing re-representation and acquisition of numerical knowledge
Source: PLoS Comput Biol. 2017 Aug 25;13(8):e1005683. doi: 10.1371/journal.pcbi.1005683 (PMC5589272; doi:10.1371/journal.pcbi.1005683)
Supplement: S1 Note — (PDF) [file pcbi.1005683.s001.pdf]

Supporting information

S1 Note **Proof** There exists a homomorphism h^* from the semigroup generated by $\{ab, ba\}$ with the operation "append" to the one generated by$\{pqr, rqp\}$ with the same operation. This homomorphism extends h' and satisfies $h^* \circ \sigma = \sigma \circ h^*$. Since the operation "symmetry" satisfies $\sigma(\lambda(x_1, x_2)) = \lambda(\sigma(x_2), \sigma(x_1))$, h^* restricted to the domain generated by $\{ab\}$ is the homomorphism h we are looking for.
