# Supplementary material for: Towards a category theory approach to analogy: Analyzing re-representation and acquisition of numerical knowledge
Source: PLoS Comput Biol. 2017 Aug 25;13(8):e1005683. doi: 10.1371/journal.pcbi.1005683 (PMC5589272; doi:10.1371/journal.pcbi.1005683)

Supporting information

S2 Note In order to shed some more light on the formalization presented in Example 0.3, we present here an alternative approach that formalizes such example by focusing on the the structure that is being preserved in the involved analogy. To this aim, we shall need the formal notion of a span. In any category **C**, a *span* from an object X to an object Y is a diagram of the form

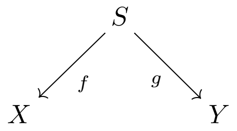

where S is an object of the category and f and g are morphisms in **C**. Note that a span with $S = X$ and $f = 1_X$ is just a morphism from X to Y, while a span with $S = Y$ and $g = 1_Y$ is a morphism from Y to X. Hence, a span can be thought of as a generalization of a morphism whose directionality is not given a priori. This makes a span useful for generalizing binary relations.

In Example 0.3, the relation between *abab* and *abba* is given by both letter-strings being generated from the same element *ab* through the operations f_s and f_t. This relation is captured by the span depicted at the left side of the diagram below. In this case, we conceptualize the source domain (A, Π) as a category whose objects are the elements of A. And for every element $x \in A$, there are exactly two morphisms with x as a source: $x \to f_t(x, x)$ and $x \to f_s(x, x)$. The right side of the diagram depicts the target domain (B, Π) as a category in a similar fashion. The dotted arrows make the entire diagram commute and represent a mapping that preserves the structure between the two categories (i.e. a functor). In this manner, the analogy is presented here as the preservation of the binary relation captured through the formal notion of a span.

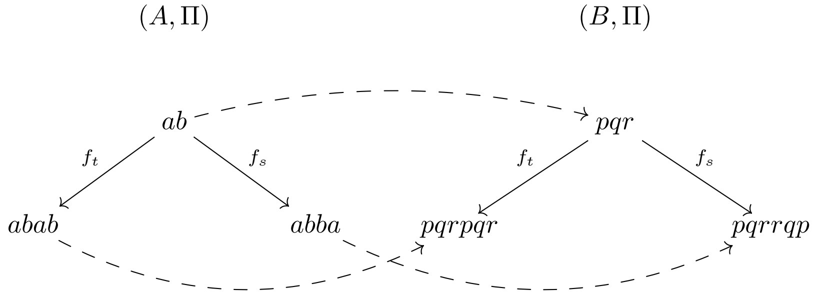

Supplement: S2 Note — (PDF) [file pcbi.1005683.s002.pdf]
