# Supplementary material for: Towards a category theory approach to analogy: Analyzing re-representation and acquisition of numerical knowledge
Source: PLoS Comput Biol. 2017 Aug 25;13(8):e1005683. doi: 10.1371/journal.pcbi.1005683 (PMC5589272; doi:10.1371/journal.pcbi.1005683)
Supplement: S3 Note — (PDF) [file pcbi.1005683.s003.pdf]

Supporting information

S3 Note **Proof** Let P be the set of all non-null strings composed from letters $a, b, ..., z$. Observe that P is a semigroup when the operation "append" is considered. It is easy to show that there exists a semigroup automorphism $h_s : P \to P$ which extends h'. Because of the definition of Π, it is easy to see that the restriction of h_s to $A \subseteq P$ is the map we need.
