# Supplementary material for: Towards a category theory approach to analogy: Analyzing re-representation and acquisition of numerical knowledge
Source: PLoS Comput Biol. 2017 Aug 25;13(8):e1005683. doi: 10.1371/journal.pcbi.1005683 (PMC5589272; doi:10.1371/journal.pcbi.1005683)
Supplement: S4 Note — (PDF) [file pcbi.1005683.s004.pdf]

Supporting information

S4 Note **Proof** Let P be the semigroup of all non-null strings composed from letters $a, b, ..., z$. Consider the semigroup automorphism $h_s : P \to P$ that extends the following map of generators:
$h_s(a) = k, h_s(b) = j, \ldots, h_s(k) = a, h_s(l) = z, \ldots, h_s(y) = m, h_s(z) = l$. Consider also the bijection $\sigma : P \to P$ that assigns to every string s its symmetric e.g. $\sigma(ijk) = kji$. The composite $h = \sigma \circ h_s$ is not a semigroup homomorphism, but it is the F-homomorphism we are looking for.
