# Supplementary material for: Towards a category theory approach to analogy: Analyzing re-representation and acquisition of numerical knowledge
Source: PLoS Comput Biol. 2017 Aug 25;13(8):e1005683. doi: 10.1371/journal.pcbi.1005683 (PMC5589272; doi:10.1371/journal.pcbi.1005683)
Supplement: S1 Appendix — (PDF) [file pcbi.1005683.s005.pdf]

Supporting information

S5 Appendix: Preliminaries for the Theorem of Re-representation

The notation used in the manuscript was aimed to improving the conceptual understanding of our model of analogy. However, in order to analyze its formal properties, we use here a slightly different notation that eases the task of applying tools provided by First Order Languages and Unification Theory. We now introduce such notation and some basic definitions.

First Order Languages

A First Order Language $\mathcal{L}^S$ is determined in a standard way (see [94, 95]) by giving a symbol set S. The symbol set S is the disjoint union of a countable set of variables, a set of function symbols, a set of relation symbols and a set of logical connectives. Throughout this work we assume that all such languages are built over the same set $V = \{x_1, x_2, x_3, ...\}$ of variables. The set of terms of $\mathcal{L}^S$ is defined as the minimal set $Term(\mathcal{L}^S)$ which satisfies:

1. if $x \in V$, then $x \in Term(\mathcal{L}^S)$, and
2. for any $n \in \mathbb{N}$, if $f \in S$ is an n-ary function symbol and $t_1, ..., t_n \in Term(\mathcal{L}^S)$, then $f(t_1, ..., t_n) \in Term(\mathcal{L}^S)$.

Since we are not going to deal with formulae here, it is not necessary to take into account the set of relation symbols and the set of logical connectives. A mapping $\rho : V \to Term(\mathcal{L}^S)$ is called a *substitution* if the set $\{v \in V \mid v \neq \rho(v)\}$ is finite. We denote a substitution by $\rho = \{v_1 \leftarrow t_1, v_2 \leftarrow t_2, ..., v_n \leftarrow t_n\}$ where $\rho(v_i) = t_i \neq v_i$ for $i = 1, ..., n$ and $\rho(v) = v$ otherwise. Observe that ρ can always be extended in a unique way to a mapping $\overline{\rho} : Term(\mathcal{L}^S) \to Term(\mathcal{L}^S)$ by defining $\overline{\rho}(t)$ as the term obtained by simultaneously replacing in t each occurrence of v_i for t_i. Sometimes, we shall write $t\rho$ instead of writing $\overline{\rho}(t)$, or $t\dfrac{t_1 t_2 ... t_n}{v_1 v_2 ... v_n}$ if we wish to make the substitution explicit. The usual composition of mappings shall be denoted by "$\circ$" and then, for example, $\overline{\rho} \circ \rho$ denotes a new substitution obtained by composition. Let $\Pi \subseteq Term(\mathcal{L}^S)$ be a set of terms, we shall say "ρ in Π" when $(Im(\rho) \setminus V) \subseteq \Pi$, where $Im(\rho)$ is the image of ρ.

Definition 0.6. *Given a non empty set* $\Pi \subseteq Term(\mathcal{L}^S)$*, the set of terms* generated by Π *is defined as the minimal set* Π^* *which satisfies the two following conditions:*

1. *If* $x \in V$*, then* $x \in \Pi^*$*.*
2. *For any substitution* ρ *in* Π^* *, if* $t \in \Pi$ *then* $t\rho \in \Pi^*$*.*

It is worth observing that for any Π, $V \subseteq \Pi^*$. Additionally, if $\Pi = V_0 \subseteq V$, then $\Pi^* = V$. In what follows, let $\mathcal{L}^S$ and $\mathcal{L}^T$ be two first order languages, interpreted as the "source language" and the "target language" respectively.

Statement of the Extensibility Problem

Let us consider $\Pi \subseteq Term(\mathcal{L}^S)$ and $\Psi \subseteq Term(\mathcal{L}^T)$ two sets without variables (i.e. $\Pi \cap V = \Psi \cap V = \emptyset$).

Definition 0.7. *A mapping* $F : \Pi \to \Psi$ *which preserves variables is called a* term translation*, i.e. for all* $t \in \Pi$

$$V(t) = V(F(t)) \tag{21}$$

where $V(t)$ *denotes the set of variables which occur in the term* t*.*

Example 0.8. *Let* $S = \{+, \cdot, 0, 1\}$ *and* $T = \{\cup, \times, \emptyset, a\}$ *be two symbol sets. Consider* $\Pi = \{0, 1, x_1 + x_2, x_3 \cdot x_4, x_1 + (x_3 \cdot x_4)\}$ *and* $\Psi = \{a \times \emptyset, a, x_1 \cup x_2, (x_3 \times x_4) \cup x_3, x_1 \cup (x_3 \cup x_4)\}$. *The bijective mapping* $F : \Pi \to \Psi$ *which assigns the i-th element of* Ψ *to the i-th element of* Π *(considering the order in which the elements of sets were listed) satisfies the conditions to be called a term translation.*

Definition 0.8. *We say that a term translation* $F^* : \Pi^* \to \Psi^*$ *is a* term morphism from Π^* to Ψ^* *when it satisfies the following two conditions:*

1. *If* $x \in V$*, then* $F^*(x) = x$.
2. *If* $t' \in \Pi$ *and* ρ *is a substitution in* Π^**, then*

$$F^*(t'\rho) = F^*(t')(F^* \circ \rho). \tag{22}$$

We now intend to characterize the existence of a term morphism $F^* : \Pi^* \to \Psi^*$ which extends a given term translation $F : \Pi \to \Psi$. Notice that for such morphism, equation (22) can be rewritten as:

$$F^*(t'\rho) = F(t')(F^* \circ \rho) \tag{23}$$

It is easy to find examples where a term translation $F : \Pi \to \Psi$ can not be extended by any term morphism. The following proposition, which can be proved by straightforward computations, shows that a term morphism F^* which extends a term translation F is characterized by its effect on all those substitutions ρ such that $Im(\rho) \subseteq \Pi^*$.

Proposition 0.3. *A term morphism* $F^* : \Pi^* \to \Psi^*$ *extends a term translation* $F : \Pi \to \Psi$ *if and only if for any substitution* ρ *in* Π^*, $\gamma = F^* \circ \rho$ *is a substitution in* Ψ^* *and makes the following diagram commute:*

$$\begin{array}{ccc} \Pi & \xrightarrow{F} & \Psi \\ \downarrow{\scriptstyle \overline{\rho}} & & \downarrow{\scriptstyle \overline{\gamma}} \\ \Pi^* & \xrightarrow{F^*} & \Psi^* \end{array}$$

Tree Representations for Terms

Next, we introduce a representation of terms which enables us to handle different ways to build a term $t \in \Pi^*$ by using recursive substitution. We denote a *directed graph* by a pair (N, E) where N is the set of *nodes* and E is the set of *arcs*. A *rooted directed tree* is a directed graph $G = (N, E)$ which is connected, it has no cycles and there exists a special node r called *root* from which all *paths* of the graph are directed. By vu we shall mean the arc which begins at node v and ends at node u. The set $\delta(v) = \{vu \in E \mid u \in N\}$ will be the set of arcs which begin at node v.

Definition 0.9. *Let* $\Pi \subseteq Term(\mathcal{L}^S) \setminus V$ *be a non empty set. A* tree over Π *is a 3-tuple* (G, ν, e) *where* $G = (N, E)$ *is a rooted directed tree,* $\nu : N \to \Pi \cup V$ *and* $e : E \to V$ *are two mappings such that for any node* $v \in N$:

- *if* $\nu(v) \in V$ *then* $\delta(v) = \emptyset$, *and*
- $\nu(v) \in \Pi$ *if and only if* $|\delta(v)| = |V(\nu(v))|$ *and* $e(\delta(v)) = V(\nu(v))$.

Given (G, ν, e) a tree over Π, we shall often refer to $\nu(v)$ as the "label" of the node v. Analogously, we shall speak of the "label" on the arc uv, meaning $e(uv)$. With this terminology, a tree over Π is a rooted directed tree (N, E) with labels, such that every node $v \in N$ has only two alternatives (see Fig. 8):

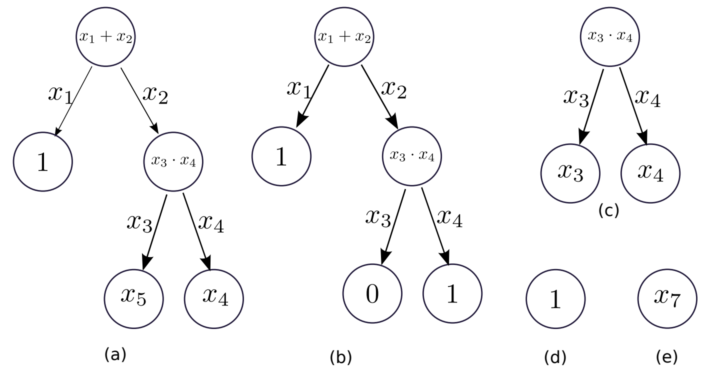

Fig 8. (a), (b) and (c) are proper trees, (c) is a fundamental tree, (d) and (e) are atomic trees, for $\Pi = \{0, 1, x_1 + x_2, x_3 \cdot x_4\}$.

1. if the label of v is a variable, it must be a leaf.
2. if the label of v is a non variable term $t \in \Pi$, then (and only then) the arcs steming from v are in 1-1 correspondence to the variables of t, via their labels.

When Π is clear from the context, we shall say "a tree" instead of "a tree over Π". A tree which has only one node (the root node) is called *atomic*, otherwise it is called *proper*. Notice that for any atomic tree, the label of its only node is a variable or it is a term without variables. In the case of a proper tree, the same condition is true for any leaf:

- v is a leaf if and only if its label is a variable or it is a term without variables.

A proper tree is *fundamental* if every arc rv (which begins at the root node) satisfies $e(rv) = \nu(v)$. Observe that fundamental trees have only two levels. The root r is at first level, labeled by a term $t \in \Pi$, and each variable of t giving rise to a leaf in the second level (see Fig. 8 (c)).

Next, we give some definitions relating trees with terms in Π^*. Let a be a tree, we shall say that $x_1, ..., x_n$ are variables of a, for some $x_1, ...x_n \in V$, if and only if $x_1, ..., x_n$ are labels of some leaves of a. Let a' be a tree with variables $x_1, ..., x_n$ and let $a_1, ...a_n$ be trees. We denote by $a' \frac{a_1 ... a_n}{x_1 ... x_n}$ the tree which results from replacing every leaf with label x_i of a' by the tree a_i.

Define the set Π° as the set of all trees over Π. Observe that every proper tree $a \in \Pi^\circ$ can be written as $a = a' \frac{a_1 ... a_n}{x_1 ... x_n}$ where a' is a fundamental tree and $a_1, ..., a_n$ are trees. To see this, take a' as the fundamental tree determined by the node r (the root of a) and the edge set $\delta(r)$, while $a_1, ..., a_n$ are the trees determined by the $n = |\delta(r)|$ children of r. We say that $a', a_1, ..., a_n$ is the *decomposition* of a. We have just sketched the proof of the following proposition:

Proposition 0.4. *Every $a \in \Pi^\circ$ is atomic or can be written as $a = a' \frac{a_1 ... a_n}{x_1 ... x_n}$, where a' is a fundamental tree and $a_1, ..., a_n$ are trees.*

This proposition will enable us to use induction to prove statements about elements of Π° by defining the *inductor* of $a \in \Pi^\circ$:

$$inductor(a) = \begin{cases} 0 & \text{if } a \text{ is fundamental,} \\ length(a) & \text{otherwise.} \end{cases}$$

where $length(a)$ is the length of the longest path in a. We can apply induction by showing first that a statement holds for any tree with inductor 0 (i.e. the atomic and

fundamental trees). And then the inductive step can be performed by observing that in any tree $a = a' \frac{a_1 ... a_n}{x_1 ... x_n}$, the inductor of a' is zero and $inductor(a_i) < inductor(a)$ for $i \in \{1, ..., n\}$.

Let us link the above paragraphs to terms. First, we are goinghow to set a url in references with latex bibliography to define a "projection" map $\pi : \Pi^\circ \to \Pi^*$ in the following inductive way:

$$\pi(a) = \begin{cases} \nu(r), & \text{if } a \text{ is atomic or fundamental,} \\ \pi(a') \dfrac{\pi(a_1)...\pi(a_n)}{x_1...x_n}, & \text{otherwise.} \end{cases}$$

where $a', a_1, .., a_n$ is the decomposition of a. It is straightforward that this mapping is well defined and surjective.

Let us now introduce some concepts for trees which are analogous to concepts introduced for terms. A *tree-substitution* in Π° is a function $\lambda : V \to \Pi^\circ$ such that the set $\{\lambda(x) \mid x \in V,\ \pi(\lambda(x)) \neq x\}$ is finite. In analogy with term substitutions we are going to denote λ as $\{x_1 \leftarrow a_1, ..., x_n \leftarrow a_n\}$ where $a_i = \lambda(x_i)$ for $i \in \{1, ..., n\}$ and if $x \neq x_i$ for all $i \in \{1, ..., n\}$, then $\pi(\lambda(x)) = x$. A tree-substitution $\lambda = \{x_1 \leftarrow a_1, ..., x_n \leftarrow a_n\}$ can be applied to a tree a, meaning that any leaf of a labeled by x is replaced by the tree $\lambda(x)$. The tree obtained in this way shall be denoted by $a\lambda$, or $a \frac{a_1 ... a_n}{x_1 ... x_n}$ if we wish to make the tree-substitution explicit. The following property is straightforward.

Lemma 0.5. *Let $a' \in \Pi^\circ$ be a tree and $\lambda = \{x_1 \leftarrow a_1, ..., x_n \leftarrow a_n\}$ be a tree-substitution in Π°, then*

$$\pi(a' \frac{a_1...a_n}{x_1...x_n}) = \pi(a') \frac{\pi(a_1)...\pi(a_n)}{x_1...x_n}.$$

From this lemma, it is clear that if F^* is a term morphism which extends a term translation F, and $a', a_1, ..., a_n$ is the decomposition of a, then:

$$F^*(\pi(a' \frac{a_1...a_n}{x_1...x_n})) = F(\pi(a')) \frac{F^*(\pi(a_1))...F^*(\pi(a_n))}{x_1...x_n}. \tag{24}$$

Characterization of Extensibility

Definition 0.10. *Let $F : \Pi \to \Psi$ be a term translation. The* tree map associated to F *is the mapping denoted by $F^\circ : \Pi^\circ \to \Psi^\circ$ which takes $a = (G, \nu, e) \in \Pi^\circ$ and changes the node labels of the tree by:*

$$F^\circ(a) = (G, F' \circ \nu, e) \tag{25}$$

where $F' : \Pi \cup V \to \Psi \cup V$ is the extension of F that acts as the identity on variables.

Thus, F° takes a tree $a \in \Pi^\circ$ and changes the label $t \in \Pi \cup V$ of each node to the label $F(t) \in \Psi \cup V$, transforming a into a tree belonging to Ψ° (see Fig. 9). From the above definition, the next lemma follows in a straightforward way.

Lemma 0.6. *Let $a \in \Pi^\circ$. If $a = a' \frac{a_1...a_n}{x_1...x_n}$, then $F^\circ(a) = F^\circ(a') \frac{F^\circ(a_1)...F^\circ(a_n)}{x_1...x_n}$.*

It is worth noticing that $F^\circ(a)$ is a well defined tree over Ψ for every $a \in \Pi^\circ$. Therefore, (unlike F^*) the tree map F° always exists. When F^* does exist, the next result describes its relation with F°.

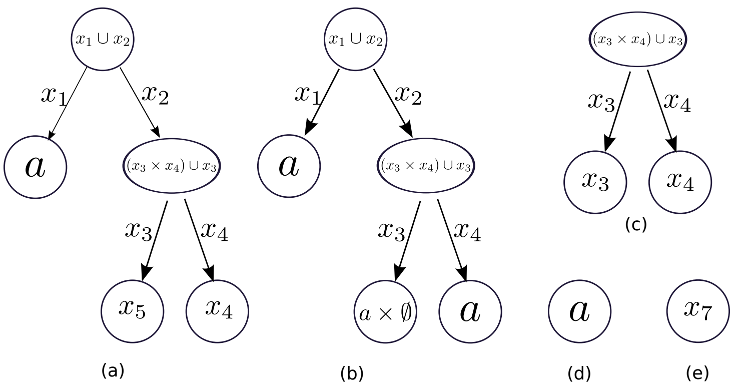

Fig 9. Outcomes of the tree map F° associated to F (from example 0.8) when it is applied on trees depicted in figure 8.

Lemma 0.7. *Let $F : \Pi \to \Psi$ be a term translation. A map $F^* : \Pi^* \to \Psi^*$ is a term morphism which extends F if and only if $F^* \circ \pi = \pi \circ F^\circ$ i.e. the following diagram commutes:*

$$\begin{array}{ccc} \Pi^\circ & \xrightarrow{F^\circ} & \Psi^\circ \\ \downarrow{\scriptstyle \pi} & & \downarrow{\scriptstyle \pi} \\ \Pi^* & \xrightarrow[F^*]{} & \Psi^* \end{array} \tag{26}$$

Proof. We use induction to prove that diagram 26 commutes necessarily when F^* is a term morphism. Let us take $a \in \Pi^\circ$. If $inductor(a) = 0$, a is an atomic or a fundamental tree. In either case

$$F^*(\pi(a)) = F^*(\nu(r)) = F(\nu(r)) = \pi(F^\circ(a)).$$

To perform the inductive step, let us assume that $inductor(a) = k > 0$ and that the condition follows necessarily for every tree of inductor less than k. Let $a', a_1, ..., a_n$ be the decomposition of a. Then:

$$\begin{aligned} F^*(\pi(a)) &= F^*(\pi(a')\frac{\pi(a_1)...\pi(a_n)}{x_1...x_n}) \\ &= F(\pi(a'))\frac{F^*(\pi(a_1))...F^*(\pi(a_n))}{x_1...x_n} \end{aligned}$$

since F^* is a term morphism extending F. Using the induction hypotheses, the last expression is equal to

$$\begin{aligned} \pi(F^\circ(a'))\frac{\pi(F^\circ(a_1))...\pi(F^\circ(a_n))}{x_1...x_n} &= \pi(F^\circ(a'\frac{a_1...a_n}{x_1...x_n})) \\ &= \pi(F^\circ(a)). \end{aligned}$$

Let us prove that the commutativity of diagram 26 is enough to ensure that F^* is a term morphism extending F. That F^* is a term translation extending F follows directly from the definition of F° and the commutation of diagram 26. Let us show that F^* is a term morphism. Take $t \in \Pi^*$ such that $t = t'\alpha$ for some $t' \in \Pi$ and $\alpha = \{x_1 \leftarrow t_1, ..., x_n \leftarrow t_n\}$ in Π^*. Take $a', a_1, ..., a_n \in \Pi^\circ$ where a' is fundamental and

such that $\pi(a') = t'$ and for $i \in \{1, .., n\}$, $\pi(a_i) = t_i$. Consider the following computations:

$$\begin{aligned} F^*(t) = F^*(t'\alpha) = F^*(\pi(a' \frac{a_1, ..., a_n}{x_1, ..., x_n})) \\ = \pi(F^\circ(a' \frac{a_1, ..., a_n}{x_1, ..., x_n})) \\ = \pi(F^\circ(a')) \frac{\pi(F^\circ(a_1)), ..., \pi(F^\circ(a_n))}{x_1, ..., x_n} \\ = F(\pi(a')) \frac{F^*(\pi(a_1)), ..., F^*(\pi(a_n))}{x_1, ..., x_n} \\ = F(t') \frac{F^*(t_1), ..., F^*(t_n)}{x_1, ..., x_n} = F(t')(F^* \circ \alpha). \end{aligned}$$

Therefore, F^* is a term morphism extending F. □

The uniqueness of F^*, when it exists, comes as a straightforward corollary of this lemma, given that the map π is surjective.

Proofs of Lemma 0.1 and Theorem of Re-representation

Lemma. *Let $F : \Pi \to \Psi$ be a term translation, $h : (A, \Pi) \to (B, \Psi)$ an F-homomorphism and $\alpha' : V \longrightarrow A$ an assignment of variables. If F^* is a term morphism that extends F, α is the extension of α' and β is the extension of $h \circ \alpha'$, then the following diagram commutes.*

$$\begin{array}{ccc} (\Pi^*, \Pi) & \xrightarrow{F^*} & (\Psi^*, \Psi) \\ \downarrow \alpha & & \downarrow \beta \\ (A, \Pi) & \xrightarrow[h]{} & (B, \Psi) \end{array}$$

Proof. To show that the above diagram commutes, let us consider Diagram 27 below. By Lemma 0.7, the rectangle at the top of Diagram 27 commutes. In order to imply that the rectangle at the bottom of the diagram commutes, we first prove that the larger rectangle commutes by using induction on the inductor of a tree in Π°. Let us take a tree a_t in Π° and set $\pi(a_t) = t$. The base step (when $inductor(a_t) = 0$) can be easily proven because the equality $h \circ \alpha(t) = \beta \circ F(t)$ is true for every term $t \in V \cup \Pi$. The inductive hypothesis assumes that $h \circ \alpha \circ \pi(a_t) = \beta \circ \pi \circ F^\circ(a_t)$ is true for every tree whose inductor is lesser than k. The computations below show that the equality holds for any tree whose inductor is equal to k.

$$\begin{array}{ccc} (\Pi^\circ, \Pi) & \xrightarrow{F^\circ} & (\Psi^\circ, \Psi) \\ \downarrow \pi & & \downarrow \pi \\ (\Pi^*, \Pi) & \xrightarrow{F^*} & (\Psi^*, \Psi) \\ \downarrow \alpha & & \downarrow \beta \\ (A, \Pi) & \xrightarrow{h} & (B, \Psi) \end{array} \tag{27}$$

$$\begin{aligned}
h \circ \alpha \circ \pi(a_t) &= h \circ \alpha \circ \pi \left(a_{t'} \frac{a_1, ..., a_n}{x_1, ..., x_n} \right) \\
&= h \circ \alpha \left(t' \frac{\pi(a_1), ..., \pi(a_n)}{x_1, ..., x_n} \right) \\
&= h(f_{t'}(\alpha \circ \pi(a_1), ..., \alpha \circ \pi(a_n))) \\
&= f_{F(t')}(h \circ \alpha \circ \pi(a_1), ..., h \circ \alpha \circ \pi(a_n))) \\
&= f_{F(t')}(\beta \circ \pi \circ F^{\circ}(a_1), ..., \beta \circ \pi \circ F^{\circ}(a_n)) - - - IH \\
&= \beta \left(F(t') \frac{\pi \circ F^{\circ}(a_1), ..., \pi \circ F^{\circ}(a_n)}{x_1, ..., x_n} \right) \\
&= \beta \circ \pi \circ F^{\circ} \left(a_{t'} \frac{a_1, ..., a_n}{x_1, ..., x_n} \right) \\
&= \beta \circ \pi \circ F^{\circ}(a_t)
\end{aligned}$$

Therefore, the larger rectangle in diagram (27) commutes. Since π is surjective, the rectangle at the bottom of Diagram (27) commutes. □

Theorem. *Let $F : \Pi \to \Psi$ be an extensible term translation, $h : (A, \Pi) \to (B, \Psi)$ an F-homomorphism and $\alpha' : V \longrightarrow A$ an assignment of variables. Consider the diagram below where F^* is the extension of F, α is the extension of α' and β is the extension of $h \circ \alpha'$. If α and β are surjective, then there exist domains (A', Π) and (B', Ψ),*

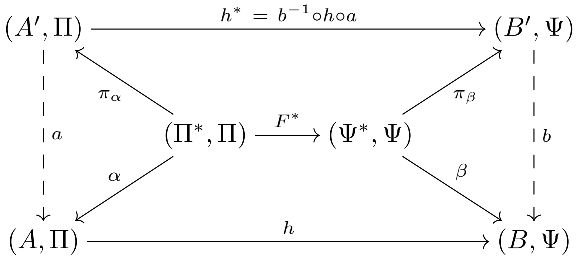

(28)

I-isomorphisms a, b and surjective I-homomorphisms π_α and π_β that make both triangles of the diagram commute. Also, $h^* = b^{-1} \circ h \circ a$ is an F-homomorphism and makes the diagram (28) commute.

Proof. The map α induces a partition on the set of terms Π^* where the equality of equivalence classes $[t_1] = [t_2]$ is determined by $\alpha(t_1) = \alpha(t_2)$. Over this set of equivalence classes, the *quotient domain of* (Π^*, Π) *with respect to* α can be defined; it is denoted by $(\Pi^*, \Pi)/\alpha$. Its key property is that the map defined by $\pi_\alpha(t) = [t]$ is a surjective I-homomorphism. Because α is surjective, the first isomorphism theorem ensures the existence of an I-isomorphism a that satisfies $a \circ \pi_\alpha = \alpha$. Therefore, by setting $(A', \Pi) = (\Pi^*, \Pi)/\alpha$, the left triangle of the diagram commutes. Also, by setting $(B', \Psi) = (\Psi^*, \Psi)/\beta$, the right triangle of the diagram commutes. By Lemma 0.1, the bottom trapezoid commutes and, finally, the entire Diagram (28) commutes by considering the composite $h^* = b^{-1} \circ h \circ a$. □
